# Molecular and epigenetic alterations in normal and malignant myelopoiesis in human leukemia 60 (HL60) promyelocytic cell line model

**DOI:** 10.3389/fcell.2023.1060537

**Published:** 2023-02-02

**Authors:** Jhinuk Basu, Swati Madhulika, Krushna Chandra Murmu, Smrutishree Mohanty, Priyanka Samal, Asima Das, Soumendu Mahapatra, Subha Saha, Indranil Sinha, Punit Prasad

**Affiliations:** ^1^ Chromatin and Epigenetics Unit, Institute of Life Sciences, Bhubaneswar, India; ^2^ RCB, Regional Centre for Biotechnology, Faridabad, India; ^3^ IMS and SUM Hospital, Siksha ‘O' Anusandhan University, Bhubaneswar, India; ^4^ Department of Obstetrics and Gynecology, KIMS, Bhubaneswar, India; ^5^ Kalinga Institute of Industrial Technology (KIIT), School of Biotechnology, Bhubaneswar, India; ^6^ Childhood Cancer Research Unit, Department of Women’s and Children’s Health, Karolinska Institutet, Solna, Sweden

**Keywords:** HL60, chromatin, transcriptome, normal myeloid differentiation, acute myeloid leukemia (AML)

## Abstract

*In vitro* cell line model systems are essential in supporting the research community due to their low cost, uniform culturing conditions, homogeneous biological resources, and easy experimental design to study the cause and effect of a gene or a molecule. Human leukemia 60 (HL60) is an *in-vitro* hematopoietic model system that has been used for decades to study normal myeloid differentiation and leukemia biology. Here, we show that IMDM supplemented with 20% FBS is an optimal culturing condition and induces effective myeloid differentiation compared with RPMI supplemented with 10% FBS when HL60 is induced with 1α,25-dihydroxyvitamin D3 (Vit D3) and *all-trans* retinoic acid (ATRA). The chromatin organization is compacted, and the repressive epigenetic mark H3K27me3 is enhanced upon HL60-mediated terminal differentiation. Differential gene expression analysis obtained from RNA sequencing in HL60 cells during myeloid differentiation showed the induction of pathways involved in epigenetic regulation, myeloid differentiation, and immune regulation. Using high-throughput transcriptomic data (GSE74246), we show the similarities (genes that did not satisfy |log2FC|>1 and FDR<0.05) and differences (FDR <0.05 and |log2FC|>1) between granulocyte-monocyte progenitor vs HL60 cells, Vit D3 induced monocytes (vMono) in HL60 cells vs primary monocytes (pMono), and HL60 cells vs leukemic blasts at the transcriptomic level. We found striking similarities in biological pathways between these comparisons, suggesting that the HL60 model system can be effectively used for studying myeloid differentiation and leukemic aberrations. The differences obtained could be attributed to the fact that the cellular programs of the leukemic cell line and primary cells are different. We validated several gene expression patterns for different comparisons with CD34^+^ cells derived from cord blood for myeloid differentiation and AML patients. In addition to the current knowledge, our study further reveals the significance of using HL60 cells as *in vitro* model system under optimal conditions to understand its potential as normal myeloid differentiation model as well as leukemic model at the molecular level.

## Introduction

Myelopoiesis generates mature myeloid cells such as monocytes, granulocytes and dendritic cells from hematopoietic stem/progenitor cells (HSPCs), and the process is tightly regulated by complex crosstalk between transcription and epigenetic factors (Ding et al., 2021). A coordinated induction of lineage-committed transcription factors with reading, writing or erasing epigenetic marks and 3D-chromatin architecture govern cell fate (Schonheit et al., 2015). Misregulation of any of the above molecular events results in differentiation blockage and unregulated proliferation of clonogenic AML blast cells, leading to malignant myelopoiesis. *In vitro* monoclonal cell lines are popular model systems for assessing the underlying molecular mechanisms of normal/malignant conditions and therapeutic drug discovery programs (Goodspeed et al., 2016; Mirabelli et al., 2019). The initial cell line-based studies provide a proof of concept to extend the study, understand molecular mechanisms and validate them in vivo and *ex vivo* model systems. Their importance in terms of availability and unlimited supply, unlike primary samples, facilitates the addressing of specific research questions related to cellular molecular biology, immunological profiles, and toxicological limits for potential drug testing (Drexler and MacLeod, 2003). Several hundred leukemia-lymphoma cell lines have been reported to date, such as HL60, NB4, KG1a, U937, THP1, K562, Jurkat, CCRF-CEM, MOLT3, and MOLT4. Having differentiation arrested at different stages of the hematopoietic lineage and harbors chromosomal aberrations that make them suitable for targeted therapy and drug studies (Sak and Everaus, 2017). Some of these leukemic cell lines hold the capacity to potentially differentiate into different hematopoietic lineages upon treatment with inducers. For example, the THP1 and U937 cell lines can be efficiently differentiated into macrophages using phorbol 12-myristate 13-acetate (PMA), and NB4 cells can be differentiated into granulocytes using *all-trans* retinoic acid (ATRA) or dimethyl sulfoxide (DMSO) (Mendoza-Coronel and Castanon-Arreola, 2016; Xu et al., 2021). However, factors such as the culture media formulations, cell density, serum, and lack of homogeneous protocols affect the gene expression profile of cell lines. This may generate varying and heterogeneous outcomes due to changes in cellular responses, such as the transcriptome, differentiation, permeability, growth, and polarization of mitochondria (Kim et al., 2015; Kreft et al., 2015; Heger et al., 2018).

In this study, we focused on human leukemia 60 (HL60) cell line, a promyelocytic AML cell line derived from a 36-year-old female with acute myeloid leukemia categorized as acute myeloblastic leukemia with a FAB-M2 classification (Ramirez et al., 2017). It has been extensively used over the years in studies related to normal myeloid differentiation and AML research for its property of aberrant proliferation with 15–30% amplification in c-myc levels and its capacity to differentiate into mature myeloid cells upon stimulation. The HL60 cell line can be efficiently differentiated into monocytes, macrophages, and granulocytes using 1α, 25-dihydroxy vitamin D3 (Vit D3), phorbol 12-myristate 13-acetate (PMA), and *all-trans* retinoic acid (ATRA) or dimethyl sulfoxide (DMSO), respectively (Manda-Handzlik et al., 2018; Yang et al., 2019; Hou et al., 2020; Prins et al., 2021). We showed that the HL60 cell line responds differently to varying culturing conditions and that myeloid differentiation ability is compromised. We showed that IMDM supplemented with 20% FBS is optimal for culture as well as for myeloid differentiation. Under these conditions, we highlight the changes in the chromatin structure, epigenetic histone marks, and genome-wide gene expression upon monocytic and granulocytic changes. Furthermore, a comparative profile of the transcriptome of HL60 cells with GMP cells and peripheral monocytes with Vit D3-induced HL60 cells showed a modest resemblance in biological pathways, while a similar analysis with leukemic blasts and HL60 cells showed significant overlap in various biological processes. In summary, we showed molecular and epigenetic alterations induced in the HL60 promyelocytic cell line (uninduced and induced states) to effectively study normal and aberrant myelopoiesis and the importance of using optimal culturing parameters.

## Materials and methods

### Ethical considerations

Ethical permission pertaining to the cord blood samples and AML patient samples (89/HEC/19) was procured from the Institutional Ethical Committee (IEC)/Institutional Review Board (IRB) of the Institute of Life Sciences. The research committee (KIMS/RPC/12/2019) and the IEC (KIIT/KIMS//IEC/41/2019) of the Kalinga Institute of Medical Sciences (KIMS) were kind enough to provide ethical permission for the cord blood samples. Institute of Medical Sciences (IMS) and SUM Hospital Siksha ‘O’ Anusandhan University (DMR/IMS.SH/SOA/180215) provided ethical permission for the AML patient’s blood samples for research. All the AML patients and/or parents who provided their blood samples were informed, and their consent was obtained.

### *In vitro* and *ex vivo* differentiation models

The HL60 cell line was purchased from American Type Culture Collection (ATCC) and maintained either in Iscove’s Modified Dulbecco’s Medium (IMDM) media supplemented with 20% fetal bovine serum (FBS) or Roswell Park Memorial Institute (RPMI) media supplemented with 10% FBS. The culturing media always contained 1% penicillin-streptomycin (Gibco-15140–122). In both media conditions, the cells were maintained at a concentration of 0.3*10^6^ cells/ml and were split at a density of 1.0*10^6^ cells/ml. Differentiation of the HL60 cell line into monocytes and granulocytes was performed using 50 nM vitamin D3 (Vit D3, Sigma D1530) and 10 μM *all-trans* retinoic acid (ATRA, Sigma R2625), respectively, for the time specified in the figure legends. For inductions of 72 h, the media was replenished with fresh Vit D3 and ATRA after the first 48 h of incubation. HL60 cells were harvested after 72 h and processed for downstream analysis. The CD34^+^ hematopoietic stem progenitor cells (HSPCs) derived from cord blood or peripheral blood of patients with AML was cultured as described in Saha *et al.* (Apostolou and Hochedlinger, 2013) The cord blood-derived CD34^+^ HSPCs were induced by 100 ng/ml (monocyte/macrophage colony-stimulating factor) M-CSF (Prospec CYT-308) and 50 ng/ml (granulocyte colony-stimulating factor) G-CSF (Prospec, CYT-220) for monocyte/macrophage and granulocytic differentiation, respectively, in Myelocult (Stem Cell Technologies, 5150) supplemented with hydrocortisone (1 µM) for 9–15 days.

### Flow cytometry analysis and sorting of undifferentiated and differentiated HL60 cells

The differentiated and undifferentiated HL60 cells were harvested (∼1.0*10^6^ cells), washed with FACS buffer (1 mM EDTA, 2% FBS in PBS), and incubated for 15 min at room temperature with CD14-APC-H7 (BD Biosciences, 20–0149-T100), CD11b-FITC (BD Biosciences, 35–0118-T100) and isotype controls APC-H7 (BD Biosciences-61427) and FITC (BD Biosciences, 555583). For human cord blood (CB), purified CD34^+^ cells, M-CSF-differentiated cells, and G-CSF-differentiated cells were scored with CD34-PE-Cy7 (BD Biosciences, 560710), CD14-BV405 (BD Biosciences, 301833) and CD66b-FITC (BD Biosciences, 561927) antibodies, respectively. Internal FACS was performed by fixing the cells using 2% paraformaldehyde and then permeabilizing the cells for incubation with H3K4me3-Alexa Fluor 647 (CST-12064S), H3K27me3-Alexa Fluor 488 (CST-5499S) and the respective isotype controls Alexa Fluor 647 (CST-3452S) and Alexa Fluor 488 (CST-4340S) using 1X permeabilization buffer (Thermo—00833356). All flow cytometry data were acquired using BD LSR Fortessa (SORP) and analyzed using FlowJo software version 10.7.1.

The uninduced and induced (Vit D3 and ATRA) HL60 cells were stained with CD14-APC-H7 and CD11b-FITC as described above. Cells were sorted for the uninduced HL60 that were negative for CD11b-FITC and CD14-APC-H7, for the Vit D3 induction which showed a high CD14-APC-H7 and a high CD11b-FITC, and for the ATRA induction which was highly positive for CD11b-FITC and negative for CD14-APC-H7, using BD FACSAria. Uninduced cells that were negative for both CD14-APC-H7 and CD11b-FITC were used as a control. The sorted cells (∼1.0*10^6^ cells) were collected and stored in TRIzol (Thermo Scientific-15596026) for RNA extraction as described below for RNA sequencing.

### Nuclear morphology assessment by May-Grünwald giemsa staining

Induced and uninduced HL60 or cord blood cells were adhered to a glass slide using Cytospin 4 (Thermo Scientific), fixed using 100% methanol for 5 min, and washed with 2 mM phosphate buffer (pH 7.2). The cells were stained in May-Grünwald (Himedia, S039) for 5 min followed by washing with water and staining with Giemsa stain (Himedia, GS500) for 20 min followed by washing. Cells were air-dried and mounted with a coverslip using DPX solution (Sigma 06522).

### Micrococcal nuclease (MNase) assay to check chromatin accessibility

Three million cells each of the wild type, Vit D3-induced monocytes and ATRA-induced granulocyte cells were fixed with 1% formaldehyde and taken for MNase digestion. Cells were treated with MNase lysis buffer supplemented with protease inhibitor (2×) followed by MNase digestion buffer supplemented with 0.5 mM PMSF and 1 M calcium chloride. The MNase enzyme (N5386, Sigma) was used in 0.03, 0.05, and 0.1 Units to one million HL60 wild-type, Vit D3 monocytes, and ATRA granulocyte cells for 5 min at 37°C. The reaction was stopped using stop buffer (0.025 M EDTA, 0.5 M EGTA, 10% SDS, 10 mg/ml Proteinase K) supplemented with proteinase K followed by overnight incubation at 65°C. The chromatin was subjected to phenol-chloroform (Himedia, MB078) treatment and precipitated at −80°C with subsequent washing with 100% and 70% ethanol. The DNA was run on a 1.5% agarose gel to check chromatin digestion during HL60 differentiation.

### RNA isolation, cDNA synthesis, and RT‒qPCR

Total RNA was isolated using the Direct-zol RNA Miniprep Kit (Zymo Research, R2052) following the manufacturer’s protocol, and 1 μg of RNA was used to make cDNA with a High Capacity cDNA Reverse Transcription Kit from Applied Biosystems (Thermo Scientific, K1672). Gene expression levels were analyzed by real-time quantitative PCR (RT‒qPCR) using the QuantStudio6 system (Applied Biosystems). The relative expression levels were calculated using the 2^−ΔΔCt^ method for each gene using *ACTB* as the housekeeping control gene.

### RNA sequencing, acquisition of datasets from GEO, and bioinformatics data analysis pipeline

Uninduced, Vit D3, and ATRA-induced HL60 cells (two biological replicates) after 72 h of induction were used for RNA extraction and library preparation using a TruSeq™ Stranded kit from Illumina according to the manufacturer’s protocol. Quality control for the library was carried out using DNA tapestation (Agilent). The samples were sequenced using the Illumina HiSeq 2500 platform, and the data were analyzed as described in Saha *et al.* 2022 (Apostolou and Hochedlinger, 2013).

Briefly, the quality of fastq files was checked using, FastQC v.0.11.5 (http://www.bioinformatics.babraham.ac.uk/projects/fastqc/), adaptor sequences were removed using BBDuk version 37.58 and the sequences were aligned using Bowtie2 version 2.3.2. With default parameters using the bowtie2-build command from the genome reference from GENCODE genome build GRCh38. Post alignment, Picard-2.9.4 (https://broadinstitute.github.io/picard/) was used for duplicate removal. Samtools-1.4.1 was used to sort and index the bam files, BAM file to a BIGWIG conversion for IGV visualization was done using the bamCoverage function from deepTools v3.1.0. Next, featureCounts v1.5.3 from a subread-1.5.3 package (https://bioweb.pasteur.fr/packages/pack@subread@1.5.3) was used with Q = 10 as mapping quality for generation of count matrix for each comparison between the control and the treated HL60 samples. This was used as an input for batch effect removal using SVA 3.29.0, then differential gene expression analysis was done using DESeq2 version 1.14.1 (Love et al., 2014) and their statistical significance was calculated using default parameters, with a threshold of log2fold change -1.5,+1.5 and adjusted *p*-value <0.05, was considered to be statistically significant. All the statistically significant genes were considered for downstream ontology and pathway analysis using Metascape (https://metascape.org).

Datasets for normal and acute myeloid leukemia hematopoietic cells were downloaded from GSE74246 (https://www.ncbi.nlm.nih.gov/geo/query/acc.cgi?acc=GSE74246). The quality check and adaptor trimming of the sequences were done as described above. The trimmed sequences were aligned using STAR v.2.5.3a with default parameters (Dobin et al., 2013) with human hg38 genome build, gencode v21 gtf 9GRCh38. Duplicate removal, featureCount extraction with Q = 10, BAM files conversion to BIGWIG for visualization in IGV, and differential gene expression analysis was carried out as described above. The genes with read counts of ≤10 in any comparison were removed followed by count transformation and statistical analysis using DESeq2 “R”. The “*p*” values were adjusted using the Benjamini and Hochberg multiple testing correction (Haynes, 2013), and the differentially expressed genes were identified (fold change of ≥ 1.5, *p*-value < 0.05). A differential gene list was made for different comparisons and subjected to gene ontology (GO) analysis using the metascape database (https://reactome.org/). The top pathways (*p* < 0.05) were used for generating heat maps using Complexheatmap (Version 2.0.0) through unsupervised hierarchical clustering. The expression clusters were annotated based on enriched GO terms. Normalized gene expression was used to generate the boxplots with a median depicting the trends in the expression across the different conditions using ggplot2 [version 3.3.5]. Principle component analysis (PCA) was performed using PCA tools from R-Bioconductor (https://www.bioconductor.org/packages/release/bioc/html/PCAtools.html). All the data are expressed as the means ± standard deviations. All comparisons were analyzed using an unpaired Student’s t-test. *p* values ≤0.05 were considered statistically significant.

## Results

### HL60 cell culture conditions and myeloid differentiation using IMDM supplemented with 20% FBS

**FIGURE 1:**
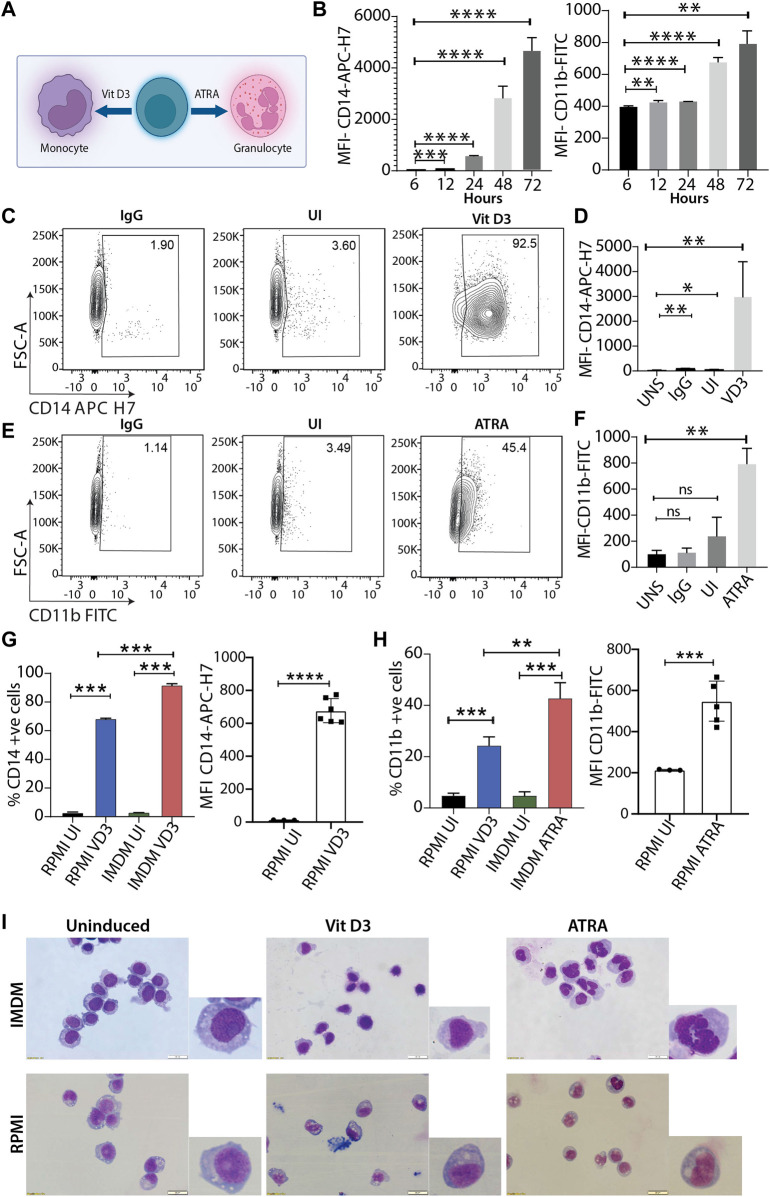
Differentiation of HL60 model system using two different culturing conditions. **(A)** Schema of HL60 myeloid differentiation model into monocytes and granulocytes using vitamin D3 (Vit D3) and *all-trans* retinoic acid (ATRA) respectively. **(B)** Bar plot shows mean fluorescence intensity (MFI) of CD14-APC-H7 and CD11b-FITC at 6, 12, 24, 48 and 72 h of induction time points with Vit D3 and ATRA (** = 0.01, *** = 0.001, **** = 0.0001). An unpaired *t*-test was performed taking a 6 h data point as a reference using GraphPad Prism version 8.2.1. **(C)** Contour plots showing gating of stained cells with IgG-APC-H7, followed by staining uninduced cells, and Vit D3 induced HL60 cells (72 h induction) with CD14-APC H7 (from left to right contour plots). The *X*-axis and *Y*-axis of the contour plots show CD14-APC H7 and forward scatter (A = area) respectively. **(D)** Bar plots representing MFI of the CD14-APC H7 intensity at 72 h post-Vit D3 induction. The MFI of CD14-APC H7 positive cells were compared with unstained cells (UNS), IgG, and uninduced cells (UI) (* = ≤0.05, ** = 0.01). An unpaired *t*-test was performed using GraphPad Prism version 8.2.1. **(E)** Similar to panel C, HL60 cells were induced with ATRA for 72 h and IgG-FITC and CD11b-FITC were used in this experiment. The *X*-axis and *Y*-axis of the contour plots show CD11b-FITC and forward scatter (A = area) respectively. **(F)** Bar plots representing MFI of the CD11b-FITC intensity at 72 h post-ATRA induction. The MFI of CD11b-FITC positive cells were compared with unstained cells (UNS), IgG, and uninduced cells (UI) (* = ≤0.05, ** = 0.01). A statistical test was performed as mentioned in panel D **(G)** Bar graphs represent the percentage of CD14 positive cells and MFI of CD14-APC H7 positive HL60 cells at 0 and 72 h in RPMI with 10% FBS and IMDM with 20% FBS. The induced cells were compared with their respective uninduced cells in both the culturing conditions in the HL60 differentiation in the IMDM and RPMI media conditions (** = 0.01, *** = 0.001). A statistical test was performed as mentioned in panel **(D) (H)** Similar to panel G but induction was carried out with ATRA for 72 h. **(I)** May Grünwald Giemsa staining showing nuclear morphology changes upon HL60 differentiation into monocytes and granulocytes in IMDM (upper panel) and RPMI (lower panel) culturing and induction conditions. The image is taken in Olympus IX83 inverted microscope using CellSens imaging software and 40× magnification.

The HL60 cell line is one of the most widely used leukemic cell lines for studying molecular mechanisms for myeloid differentiation, leukemia, drug treatment, *etc.* (Maniwa et al., 2015; Macias et al., 2018; Manda-Handzlik et al., 2018; Pan et al., 2019). Various studies in the past have shown that culture conditions affect the final experimental outcome (Kim et al., 2015; Kreft et al., 2015). Since HL60 cells were procured from ATCC, we used their recommended culturing conditions and performed a time course experiment with vitamin D3 (Vit D3) and *all-trans* retinoic acid (ATRA) inductions to identify the duration where CD14 and CD11b expression was highest for monocytes and granulocytes, respectively (Figure 1A). HL60 differentiation with Vit D3 and ATRA was carried out at 6, 12, 24, 48, and 72 h, and the levels of differentiation were scored by estimating the expression levels of CD14-APC-H7 and CD11b-FITC markers using flow cytometry (Figure 1B). We observed a major spike in the expression (represented as MFI) of CD14 levels at 48 h post-induction, and it further increased at 72 h of induction (*p* = 0.0026). However, in the case of ATRA induction, lower levels of CD11b were maintained and showed an initial spike after 24 h (*p* = <0.0001 with respect to 6 h) and reached its maximum by 72 h (*p* = 0.0012 with respect to 6 h). This gave us an overview of the HL60 differentiation kinetics, and we selected 72 h for our further studies. Next, we induced HL60 cells with both Vit D3 and ATRA and compared the levels of myeloid differentiation with unstained (UNS), IgG, and uninduced (UI) with the differentiated cells. We observed significant enhancements in the MFI of Vit D3 (Figure 1D, *p* = 0.0139 with respect to UNS) and ATRA (Figure 1F, *p* = 0.0052 with respect to UNS) stimulations with more than 90% CD14 positivity in the Vit D3 (Figure 1C) and over 40% CD11b positivity in the ATRA inductions (Figure 1E and Supplementary Figure S1A). To further confirm the HL60 differentiation patterns, May-Grünwald Giemsa staining was performed in the uninduced, Vit D3-treated, and ATRA-induced HL60 (Figure 1I). We observed round nuclear morphology in the uninduced HL60 cells, which were changed into a kidney-shaped nucleus and lobbed nucleus upon Vit D3 and ATRA induction, respectively (Figure 1I). These experiments show optimized growth and myeloid differentiation conditions of HL60 cells in ATCC-recommended culturing conditions.

### IMDM media efficiently promotes myeloid differentiation in HL60 cells compared with RPMI media

Researchers over the decades have primarily used two culturing conditions for HL60 cells, RPMI supplemented with 10% FBS and IMDM supplemented with 20% FBS. Therefore, after optimizing myeloid differentiation in ATCC-prescribed conditions, we performed experiments in HL60 cells cultured in RPMI supplemented with 10% FBS and compared the myeloid differentiation levels with IMDM culturing conditions. Interestingly, we observed an average of 68.5% CD14 positivity for Vit D3 (Figure 1G) and 24% CD11b positivity for ATRA induction (Figure 1H, left panel). We observed a 1.25- and 1.73-fold higher differentiation for Vit D3 and ATRA inductions, respectively, in the IMDM compared to the RPMI. A similar trend was observed in the MFIs of the RPMI group, where the differentiation levels were significantly reduced compared with IMDM conditions (Figures 1G,H). Although we observed significant induction of myeloid markers, nuclear morphology changes are a better indication of the extent of myeloid differentiation. Therefore, we compared May Grünwald Giemsa staining for HL60 grown and induced in RPMI and IMDM culturing conditions. The nuclear morphology showed significant changes for both Vit D3 and ATRA inductions in IMDM culturing conditions compared with RPMI. RPMI media conditions for induction showed more immature monocyte and metamyelocyte stage differentiation (Figure 1I) (Baxter et al., 2009). Cell differentiation and the cell cycle have a significant association wherein terminal differentiation is accompanied by an exit from the cell cycle (Ruijtenberg and van den Heuvel, 2016). Therefore, we next assessed the cell cycle profile of the HL60 differentiation model, where we observed significant differences between IMDM and RPMI conditions (Supplementary Figure S1B–E). These findings suggested that myeloid differentiation is induced to a lesser extent in RPMI supplemented with 10% FBS culturing conditions compared to IMDM supplemented with 20% FBS formulation, which promotes efficient myeloid differentiation.

### Myeloid differentiation induces changes in chromatin architecture and epigenetic histone modifications

**FIGURE 2:**
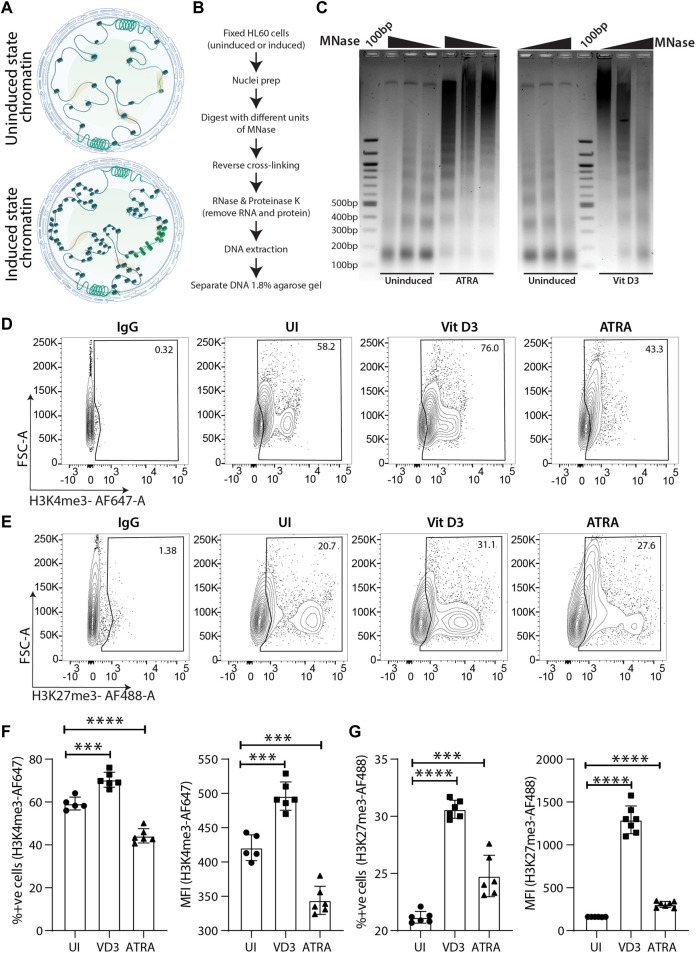
Chromatin dynamics during the course of HL60 differentiation. **(A)** Schematic representation of the chromatin changes induced in the hematopoietic stem/progenitor cells (HSPCs) upon differentiation. **(B)** Flowchart showing step wise process for performing the MNase assay. **(C)** Micrococcal nuclease (MNase) digestion shows the appearance of the nucleosomal ladder in uninduced HL60 and the lack of clear digestion pattern in HL60 induced with ATRA (left) and Vit D3 (right). **(D)** Contour plots showing gating of stained cells with IgG-AF647, uninduced cells, Vit D3 induced and ATRA induced HL60 cells for 72 h (from left to right) stained with H3K4me3-AF647. The *X*-axis and *Y*-axis of the contour plots show H3K4me3-AF647 and forward scatter (A = area) respectively. **(E)** Contour plots showing gating of stained with IgG-AF488, uninduced cells, Vit D3 induced and ATRA induced HL60 cells for 72 h (from left to right) stained with H3K27me3-AF488. The *X*-axis and *Y*-axis of the contour plots show H3K27me3-AF488 and forward scatter (A = area) respectively. **(F)** Bar plots representing the levels of percent positive cells and MFI intensity of the H3K4me3-AF647 post 72 h of Vit D3 and ATRA induction in HL60. The percent positive cells and MFI of H3K4me3-AF647 were compared with unstained cells (UNS), IgG and uninduced cells (UI) (*** = 0.001, **** = 0.0001). An unpaired *t*-test was performed using GraphPad Prism version 8.2.1. **(G)** Bar plots representing the levels of percent positive cells and MFI intensity of the H3K27me3-AF488 post 72 h of Vit D3 and ATRA induction in HL60. The percent positive cells and MFI of H3K27me3-AF488 were compared with unstained cells (UNS), IgG and uninduced cells (UI) (*** = 0.001, **** = 0.0001). All the statistical test was performed as mentioned in GraphPad Prism version 8.2.1.

Global transcriptomic changes in myeloid cells also guide chromatin architectural changes from being open in undifferentiated HSPCs to a condensed state in differentiated cells (Gaspar-Maia et al., 2011). Pluripotent stem cells undergo a series of chromatin organizational changes during differentiation upon induction (Apostolou and Hochedlinger, 2013). Generally, the open chromatin structure is compacted systematically in a manner that lineage-specific chromatin is not condensed (Bao et al., 2015; Allshire and Madhani, 2018) (Figure 2A). Therefore, we were interested in understanding whether wild-type (WT) or uninduced HL60 cells also undergo chromatin compaction upon myeloid differentiation. To this end, we performed a partial chromatin digestion assay using the micrococcal nuclease (MNase) enzyme (Figure 2B). The uninduced Vit D3-and ATRA-treated HL60 cells were digested with increasing concentrations of MNase, and the DNA extract was run on an agarose gel. The uninduced HL60 chromatin showed partial digestion of chromatin and nucleosomal ladder where the mono, di, tri, tetra, and penta nucleosomal DNA fragments could be distinguished on the agarose gel (Figure 2C). However, the resolution of the nucleosomal DNA ladder was significantly reduced, and undigested chromatin could be seen in HL60 cells induced with Vit D3 and ATRA. Such global chromatin changes in chromatin organization prompted us to assess active (H3K4me3) and repressive (H3K27me3) epigenetic histone modifications. Several researchers have used the flow cytometry method to assess global changes in histone modifications upon cell differentiation, drug treatment, *etc.* (Obier and Muller, 2010; Benedikt et al., 2011; La Noce et al., 2018). Using FACS for H3K4me3 and H3K27me3 histone modification marks, we observed a significant reduction in H3K4me3 (Figures 2D,F) and an increase in H3K27me3 levels (Figures 2E,G) in the ATRA induced cells compared to the uninduced HL60 cells. However, Vit D3-induced monocytes showed a significant increase in both active and repressive epigenetic marks, which could potentially be due to its non-terminal differentiation nature and its capability of further differentiating into macrophage cells. The global repressive mark H3K27me3 was significantly enhanced in both Vit D3 monocytes and ATRA granulocytes compared to wild-type or uninduced HL60 cells, which confirms that during HL60 differentiation, chromatin not only loses its accessibility and active histone marks but also gains repressive histone marks (Figures 2D,F). Together, myeloid differentiation drives terminal differentiation in HL60, which changes not only its overall chromatin architecture but also epigenetic marks toward a refractory state from an open and active chromatin state.

### Genome-wide gene expression changes in ATRA- and Vit D3-induced HL60 cells stimulate myeloid-specific pathways

**FIGURE 3:**
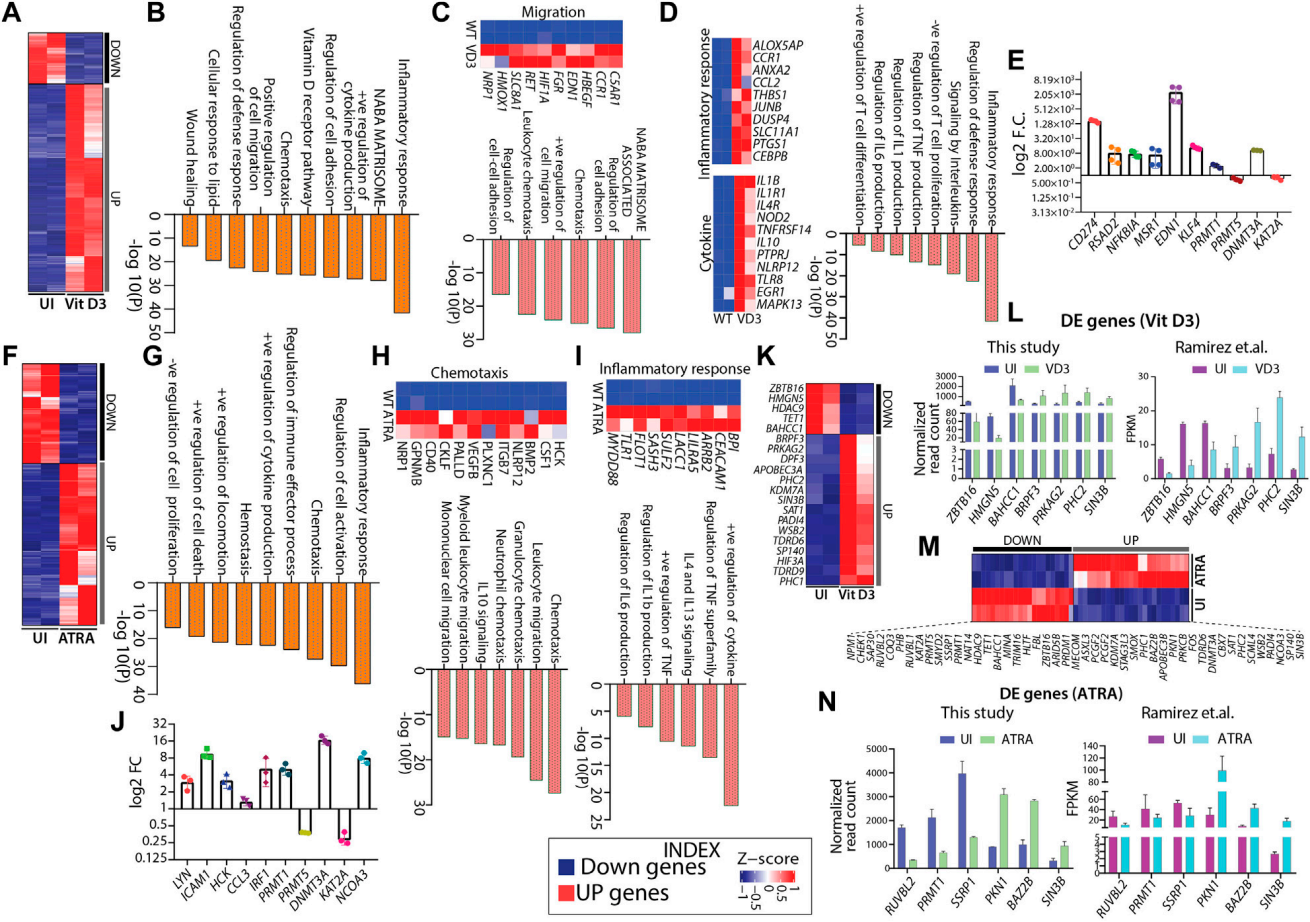
Gene expression changes in HL60 during myeloid differentiation. **(A)** Heatmap showing gene expression changes with Vit D3 induction for 72 h in HL60 cells. **(B)** Bar graph showing significance (-log10 as the *p*-value) for each pathway enriched from the upregulated genes obtained from Vit D3 induction compared with uninduced HL60 cells. **(C–D)** Heatmap and biological pathways (significance -log10 as the *p*-value) are associated with cell migration, inflammatory response, and cytokine response from the set of genes induced during Vit D3 induction. **(E)** RT**-**qPCR validation of the selected pathways like an inflammatory response (*RSAD2*, *NFKBIA*), cytokine response (*MSR1, EDN1*), cytoskeleton regulation (*CD274*, *KLF4*) and epigenetic factors (*PRMT1, PRMT5, DNMT3A, KAT2A*) in the Vit D3 monocytes compared to the uninduced HL60 cells. **(F)** Heatmap similar to panel A, except differential gene expression is from 72 h post-ATRA induction. **(G)** Bar graph showing the pathways (significance -log10 as the *p*-value) enriched from upregulated genes obtained in ATRA induction. **(H–I)** Heatmap and biological pathways associated with chemotaxis and inflammatory response from upregulated gene sets in ATRA induced HL60 cells **(J)** RT-qPCR validation of genes involved in regulating selected biological pathways such as inflammatory response and cytokine response (*IRF1, CCL3, HCK*), cell migration (*ICAM1, LYN*), and epigenetic factors (*PRMT1, PRMT5, DNMT3A, KAT2A, NCOA3*) in ATRA induced HL60 cells compared to uninduced HL60 cells. Metascape version 3.5 is used for all pathway analyses. **(K)** Heatmap showing the gene expression profile of the differentially expressed epigenetic factors with Vit D3 induction for 72 h in HL60 cells. **(L)** Bar graphs showing the normalized read count obtained from this study (*n* = 7) (left) and FPKM values obtained from the Ramirez et al. study (*n* = 7) (right) for a few epigenetic factors as obtained in panel **(K) (M)** Similar to panel K, except the HL60 cells were induced with ATRA for 72 h. **(N)** Bar graphs showing the normalized read count obtained from this study (*n* = 6) (left) and FPKM values obtained from the Ramirez et al. study (*n* = 6) (right) for a few epigenetic factors as obtained in panel M.

Hematopoietic differentiation is regulated by a complex interplay of gene regulatory programs. Therefore, we aimed to capture the genome-wide transcriptome changes associated with HL60 myeloid differentiation. We performed RNA sequencing from FACS-sorted uninduced (negative for CD11b-FITC and CD14-APC-H7, Supplementary Figure S2B), Vit D3 (double positive for CD11b-FITC and CD14-APC-H7, Supplementary Figure S2C), and ATRA-induced (highly positive for CD11b-FITC and negative for CD14-APC-H7, Supplementary Figure S2D) HL60 cells (Supplementary Figure S2A–D). Principal component analysis (PCA) showed overall relatedness between the biological replicates of induced and uninduced HL60 (Supplementary Figure S3A). Differential gene expression analysis revealed 1146 and 2115 genes in Vit D3 (Figure 3A) and ATRA-induced (Figure 3F) HL60 cells, respectively (Figures 3A,F). ATRA induction resulted in 1309 upregulated genes and 806 downregulated genes, while Vit D3 induction induced 921 genes and downregulated 225 genes (Supplementary Figure S3B). The differentially expressed genes obtained from Vit D3 and ATRA inductions (FDR ≤0.05) with log2-fold change ≥1.5 were subjected to pathway analysis using Metascape (Zhou et al., 2019). Both Vit D3 (Figure 3D) and ATRA (Figure 3I) inductions primarily induced immune response pathways with positive regulation of cytokines such as IL6, IL1, TNF, IL13, *etc.*, and activation, proliferation, and differentiation of T cells (Figures 3D,I). We also observed the enrichment of pathways related to migration, chemotaxis, leukocyte migration, regulation of cell adhesion, and the NABA matrisome, which are important for myeloid cells such as monocytes and granulocytes that perform diapedesis, phagocytosis, *etc.*, during infection in the Vit D3 (Figure 3C) and ATRA (Figure 3H) stimulations. The downregulated pathways were related to AML-related pathways along with Wnt-beta catenin, CMYB, and ras pathways which are frequently mutated in leukemic conditions (Supplementary Figure S2C). We validated genes from different pathways, such as the inflammatory response (*RSAD2*, *NFKBIA*), cytokine response (*MSR1, EDN1*), and cytoskeleton regulation (*CD274*, *KLF4*) (Figure 3E) (Kowalczyk et al., 2015; Brauer et al., 2018; Kurokawa et al., 2019; Eichberger et al., 2020; Kircheis et al., 2020; Kong et al., 2020). ATRA stimulation in HL60 cells also elicited pathways for positive regulation of cell death and negative regulation of cell proliferation, further confirming terminal differentiation (Figure 3G). Similarly, the MYC pathway, double-strand break repair, DNA replication, and cell cycle pathways were downregulated in ATRA-induced HL60 cells (Supplementary Figure S3D). Vit D3-induced HL60 cells showed enrichment of lipid metabolism, regulation of wound healing, and vitamin D receptor pathways (Figure 3B). We validated the gene expression of five genes obtained from ATRA induction using RT‒qPCR from different categories, such as inflammatory response, cytokine response, and cell migration. The fold changes in *LYN*, *ICAM1*, *HCK*, *CCL3*, and *IRF1* were significantly high, suggesting their high transcript levels in ATRA/granulocytic induction (Figure 3J) (Cougoule et al., 2010; He et al., 2011; Smolinska et al., 2011; Gibaldi et al., 2020; Song et al., 2021; Taftaf et al., 2021). These observations show that ATRA and Vit D3 induction in HL60 cells elicits transcriptional programs essential for the functions of myeloid cells such as granulocytes and monocytes.

### Differential gene expression analysis from HL60 transcriptomic revealed various epigenetic factors potentially involved in myelopoiesis

The dynamic interplay between both transcription factors and epigenetic factors are key determinants driving hematopoietic differentiation and tuning the lineage choice and cell fate of HSPCs. Earlier, we showed how HL60 differentiation into monocytes and granulocytes affected the chromatin architecture and histone modifications in undifferentiated and differentiated cells. Thus, the epigenetic landscape was rewired during HL60-mediated myeloid differentiation, which might have the potential to reprogram the differentiation process (Alvarez-Errico et al., 2015; Ivashkiv and Park, 2016). Therefore, we further mined transcriptomic data to identify differentially expressed epigenetic factors potentially playing important roles in Vit D3 and ATRA induction. We identified several epigenetic factors that play essential roles in histone acetylation, methylation, phosphorylation, demethylation, and deacetylation in Vit D3 (n = 20, Figure 3K) and ATRA inductions (*n* = 47, Figure 3M) (Figure 3K and M). For example, we observed the enrichment of ZBTB16, KDM7A, SIN3B, and BAZ2B, which were reported to regulate normal hematopoietic differentiation, and their dysregulation leads to aberrant hematopoiesis (Suliman et al., 2012; Cantor and David, 2017; Arumugam et al., 2020). The functional role of selected epigenetic factors for Vit D3 and ATRA inductions compared with uninduced HL60 is summarized in Supplementary Tables S1 and S2. Moreover, we validated a few epigenetic factors like PRMT1, PRMT5, and DNMT3A in the Vit D3 (Figure 3E) and ATRA-induced cell by qPCR (Figure 3J). Furthermore, we compared our gene expression data with the transcriptomic data from Ramirez *et al.* for both Vit D3 and ATRA inductions, where the inductions were carried out for 72 h (this study) and 96 h (Ramirez et al.) (Ramirez et al., 2017). We compared seven genes from Vit D3 (Figure 3L) induction and six genes from ATRA induction (Figure 3N) with uninduced transcriptomes and found similar gene expression profiles (Figure 3L and N). This comparative study further strengthens the role of epigenetic factors in myeloid differentiation progression, which can be further evaluated for their mechanistic role in HL60 *in vitro* and *ex vivo* myeloid differentiation models.

### A comparative profile of the transcriptome of HL60 and normal hematopoietic progenitor’s myeloid differentiation revealed overlapping and unique pathways

**FIGURE 4:**
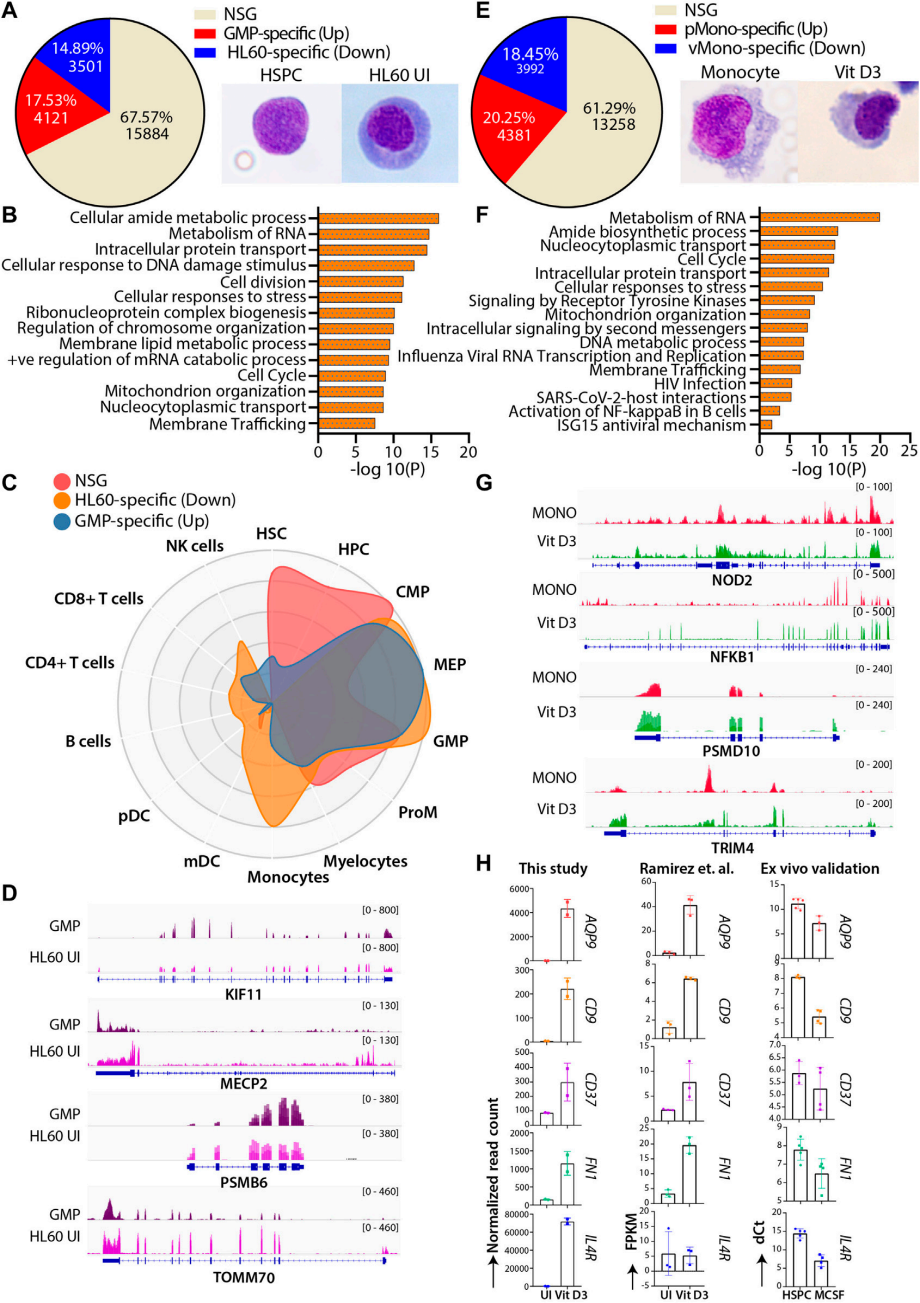
Comparative transcriptomic profiles of the HL60 (n = 2) vs GMP (n = 4) and Vit D3 induced HL60 (n = 2) vs primary monocytes (n = 4). **(A)** Venn diagram representing the differential gene expression profile of HL60 uninduced cell with GMP. The differential gene expression analysis resulted in upregulated genes (>1-fold change, 17.53%, n = 4121, adjusted *p*-value ≤0.05), downregulated genes (<1-fold change, 14.89%, n = 3501, adjusted *p*-value ≤0.05) and genes that did not satisfy |log2FC|>1 and FDR<0.05 (NSG) (67.57%, n = 15884). **(B)** Bar plots representing the NSG-specific biological pathways (significance -log10 as the *p*-value) such as cell cycle, translation, intracellular protein transport, metabolism of lipid, and metabolism of RNA conserved between the GMP and the uninduced HL60 cells. **(C)** CellRadar image representing the overall relatedness between GMP and uninduced HL60 cells. **(D)** Representative IGV plots showing the gene expression levels of the *KIF11, MECP2, PSMB6,* and *TOMM70* in GMP and uninduced HL60. **(E)** Venn diagram representing the differential gene expression profile of Vit D3 induced-monocytes with primary monocytes. Overall relatedness of the primary bone marrow monocytes with the Vit D3 induced monocytes. The differential gene expression analysis for the Vit D3-induced monocytes showed upregulated genes (>1-fold change, 20.25%, n = 4381, adjusted *p*-value≤0.05), downregulated genes (<1-fold change, 18.45%, n = 3992, adjusted *p*-value ≤0.05) and genes that did not satisfy |log2FC|>1 and FDR<0.05 (NSG) (61.29%, n = 13258). **(F)** Bar plots representing the NSG-specific pathways (significance -log10 as the *p*-value) conserved between Vit D3 monocytes and primary monocytes such as immune response, translation, nucleocytoplasmic transport, cell cycle, and cellular respiration. **(G)** Representative IGV plots showing the gene expression levels of the *NOD2, NFKBIA, PSMD10,* and *TRIM4* in primary monocytes and HL60 Vit D3 monocytes. **(H)** Bar graphs showing the normalized read count, n = 2 (left), dCt values of the RT**-**qPCR validation in the *ex vivo* cord blood-derived CD34^+^ differentiation model system (*n* = 4), and the FPKM values from Ramirez et al. study, *n* = 3 (right) of the selected genes (*CD9, AQP9, IL4R, CD37, FN1*) in the uninduced and monocytic conditions.

HL60 cells are extensively used to study normal myeloid differentiation programs; therefore, we attempted to compare the genome-wide gene expression profile of granulocyte-monocyte progenitors (GMPs) with uninduced HL60-and Vit D3-induced monocytes with primary monocytes (Maniwa et al., 2015; Manda-Handzlik et al., 2018). This comparison helped us identify the similar and unique molecular changes associated with HL60 differentiation in vitro systems and primary cells. To this end, we extracted and compared the transcriptome of bone marrow-derived GMP (GSE74246) and uninduced HL60 cells. The differential gene expression analysis was categorized into three different classes: the upregulated genes (GMP-specific), the downregulated genes (HL60-specific) with |log2FC|>1 and FDR<0.05, and a set of genes that did not satisfy |log2FC|>1 and FDR<0.05 were classified as the non-significant gene set (NSG). The NSG category was considered a set of genes with similar gene expression profiles between the groups and may have overlapping gene regulatory programs. The NSG expression profile between the GMP and uninduced HL60 showed 67.57% (*n* = 15884) of genes, while 17.53% (*n* = 4121) and 14.89% (*n* = 3501) genes were GMP-specific and HL60-specific, respectively (Figure 4A). The genes from the NSG category showed enrichment for fundamental pathways such as cell cycle regulation, translation, intracellular protein transport, and metabolism (Figure 4B). The GMP-specific genes were enriched for pathways related to transcriptional programs for hematopoietic cell lineage, regulation of cell adhesion, regulation of cytokine response, *etc.* While HL60-specific genes were enriched in transcriptional regulation of myeloid differentiation, inflammatory response, metabolic processes, transcriptional misregulation in cancer, *etc.* (Supplementary Figure S4A and S4B). To further understand the cell signature of the differential gene expression from GMP-HL60 uninduced, we subjected gene sets to the CellRadar analysis tool (Dhapola et al., 2020). Interestingly, the NSG set showed a progenitor phenotype, the GMP-specific set showed a stem/progenitor phenotype, and the HL60-specific set gave both progenitor and monocytic phenotypes (Figure 4C). We looked into IGV plots for selected genes for the NSG category of genes such as *KIF11*, *MECP2*, *PSMB6,* and *TOMM70*, which are implicated in the cell cycle, DNA and chromatin packaging, and components of proteasome and mitochondria, respectively, to show similar levels of gene expression profiles between GMP and uninduced HL60 (Figure 4D). This indicated that HL60 cells share a progenitor phenotype with respect to their transcriptional programs and are tuned for myeloid cell functions.

Using a similar approach, we compared the transcriptome of primary peripheral blood monocytes (pMono) and Vit D3-induced monocytes in the HL60 (vMono) transcriptome profile. We obtained 61.29% (*n* = 13258) of genes as the NSG, 20.25% (*n* = 4381) genes as pMono-specific, and 18.45% (n = 3992) genes as vMono-specific. (Figure 4E). The top 2500 genes from the NSG category revealed the conservation of fundamental biological functions such as immune response, translation, cellular transport, and cell cycle (Figure 4F). The upregulated or pMono-specific genes (FDR <0.05 and log2FC > 1) were associated with cytokine signaling, defense response to the virus, positive regulation of cytokine production, inflammatory response, and cell migration biological pathways (Supplementary Figure S4C), while the downregulated or vMono-specific genes (FDR ≤0.05 and log2FC < -1) were involved in functions such as cell cycle, replication, chromosome maintenance, *etc.* (Supplementary Figure S4D). This analysis suggested that pMono is more mature for its immune function than vMono and that HL60 Vit D3 induction may require either a higher concentration of Vit D3 or a longer induction time. The IGV plots for the NSG category showed similar expression patterns of *NOD2*, *NFKBIA*, *PSMD10,* and *TRIM4*, which are implicated in the inflammatory response, regulation of the cell cycle, and interferon response (Figure 4G). Furthermore, we used cord blood-derived HSPCs and induced them with M-CSF for selected gene validation (Supplementary Figure S5A and S5B). We validated selected genes, such as *AQP9*, *CD9*, *IL4R*, *CD37*, and *FN1*, which are implicated in the inflammatory response and activation of leukocyte and vitamin D pathways, and found upregulated gene expression upon M-CSF induction (Figure 4H). Our analysis found biological processes that are conserved and unique between HL60 cells and *in vivo* cells along with the comparability of *in vivo* and HL60-mediated myeloid differentiation.

### Transcriptomic of the HL60 cell line shows reasonable similarity with leukemic blasts

**FIGURE 5:**
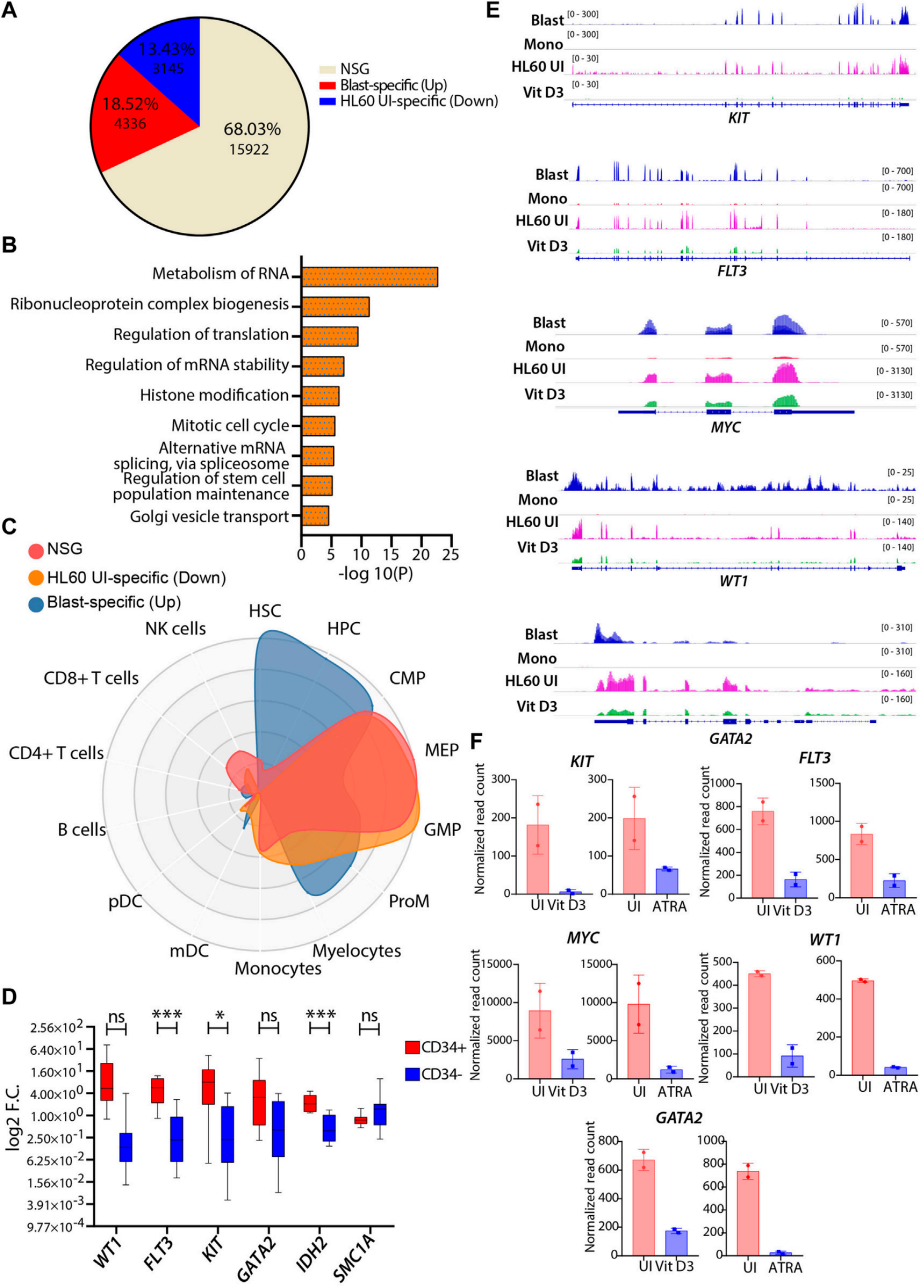
Comparative transcriptomic profiles of uninduced HL60 and leukemic blasts derived from patients with AML. **(A)** Venn diagram representing the differential gene expression profile of HL60 uninduced cell with primary AML blast. The differential gene expression analysis resulted in upregulated genes (>1-fold change, 13.43%, n = 3145, adjusted *p*-value ≤0.05), downregulated genes (<1-fold change, 18.52%, n = 4336, adjusted *p*-value ≤0.05) and genes that did not satisfy |log2FC|>1 and FDR<0.05 (NSG) (68.03%, n = 15922). **(B)** Bar plots showing the enriched pathways (significance -log10 as the *p*-value) for the NSG genes. **(C)** CellRadar plot highlighting the overall relatedness between the primary AML blast and uninduced HL60 cells. **(D)** RT**-**qPCR validations of the genes upregulated in the AML like *WT1, FLT3, KIT, GATA2, IDH2,* and *SMC1A* in the primary AML CD34 positive and negative cells. **(E)** Representative IGV plots showing the gene expression profile of the leukemia-associated genes, *KIT, FLT3, MYC, WT1,* and *GATA2* in the primary AML blast cells (n = 8), primary monocytes (n = 4), uninduced HL60 (n = 2) and Vit D3-induced monocytes (n = 2). **(F)** Bar graphs showing the normalized read count values from this study (*n* = 5) of above-mentioned leukemia-associated genes in the uninduced HL60 and Vit D3 and ATRA-induced HL60.

HL60, a leukemic cell line with high proliferative capacity and overexpression of the *MYC* oncogene, is a popular model among researchers used to study aberrant myelopoiesis. Therefore, we tried to address the transcriptional correlation of the wild-type HL60 cells with the primary AML blast cells using a similar approach described in the previous section. The differential gene expression analysis revealed that 68.03% of the genes (n = 15922) belonged to the NSG category, and the pathway analysis showed enrichment of metabolism of RNA, cell cycle, chromatin organization, intracellular transport, and metabolism-related pathways for this group (Figures 5A,B). These processes are essential for the proliferation and homeostasis of neoplastic cells such as AML blast and HL60 cells (Schnerch et al., 2012; Nix and Price, 2019; Fang et al., 2020). CellRadar analysis showed that the blast-specific genes had stem/progenitor cell signatures, while HL60-specific and NSG category genes showed progenitor-type signatures. This shows that HL60 cells share decent phenotypic characteristics compared with leukemic blasts (Figure 5C). The upregulated genes (FDR ≤0.05 and log2FC > 1) in AML blast were enriched for pathways associated with the hematopoietic cell lineage, cell adhesion, regulation of cytokine production, angiogenesis, *etc.* (Supplementary Figure S6A). The pathways that are specific to HL60 (FDR ≤0.05 and log2FC < -1) were associated with pathways such as DNA metabolic process, cell cycle, translation, and DNA repair (Supplementary Figure S6B). Together, the pathways enriched for the differential gene expression analysis were indicative of aberrant transcriptional programs in AML blast cells that could potentially contribute to its heterogeneity. Next, we identified well-characterized AML-associated oncogenic genes, such as *WT1*, *FLT3*, *KIT*, *GATA2*, *IDH2*, and *SMC1A,* and validated their gene expression in CD34^+^ cells derived from peripheral/bone marrow blood of AML patients by RT‒qPCR. We found that all patients with AML CD34^+^ cells had high expression of these genes compared with the CD34-negative population obtained from the same patients (Figure 5D). Similar observations were verified in gene expression datasets from GSE74246 (Figure 5E). Apart from this, we also checked the transcript levels of *KIT*, *FLT3*, *MYC*, *WT1,* and *GATA2*, which were high in the uninduced HL60 and were significantly reduced upon Vit D3 and ATRA induction (Figure 5F). This indicates that the induction of myeloid differentiation results in the rewiring of the gene regulatory program and that the leukemic burden could be reduced. In conclusion, we observed a reasonable correlation between the transcriptional profiles of HL60 cells and leukemic blast cells.

## Discussion

Human leukemia 60 (HL60) cell line is commonly used to study normal and aberrant myelopoiesis compared with other leukemic cell lines due to its myeloid differentiation ability. Vit D3 and ATRA induce HL60 cells to differentiate into monocytes and granulocytes and stimulate lineage-specific genes such as CD14 and CD11b, respectively. Due to the discrepancy in the culturing conditions for HL60 by different research groups, we optimized the culturing and myeloid differentiation in IMDM supplemented with 20% FBS. We also showed the epigenetic changes and genome-wide gene expression changes incurred during myeloid differentiation. Since HL60 cells have been used as a model system to study both normal and aberrant myelopoiesis, we compared the transcriptomes of GMP vs uninduced HL60, pMono vs vMono, and leukemic blasts vs uninduced HL60. This comparison not only revealed differences between these subsets but also similar properties that potentially make HL60 a suitable model for studying myelopoiesis.

Open chromatin structure is a hallmark of stem cells with reduced heterochromatin levels, which has been studied in both *in vitro* and *in vivo* model systems (Gaspar-Maia et al., 2011; Schlesinger and Meshorer, 2019). The open chromatin state is essential for a stem/progenitor cell to imbibe signals for lineage commitment and differentiation. Lineage commitment is maintained by several chromatin modulators that selectively condense chromatin regions that are not essential for lineage progression and differentiation. Similarly, we showed a global open chromatin state in the uninduced state of HL60, which further condensed upon myeloid differentiation. Epigenetic alterations such as posttranslational histone modifications, chromatin remodeling, changes in chromatin conformation, and DNA methylation are documented in myeloid development (Schonheit et al., 2015). Terminal differentiation is accompanied by pursuing the gene expression of selected candidate genes essential to perform the said function. For example, the terminally differentiated granulocytes are tuned to maintain the expression of elastase, proteinase 3, *etc.*, which are important for innate immune function (Korkmaz et al., 2010). Moreover, the cell would not use its limited resources for energy-consuming gene expressions that are not essential for their function. These modulations are carried out effectively by epigenetic modifications as well as the spatial temporal arrangement of chromatin (Schonheit et al., 2015). The ATRA induction, resulting in terminally differentiated granulocytes in HL60, showed reduced active H3K4me3 marks and an increase in repressive H3K27me3 marks, showing the shutting down of transcriptional programs. However, proliferating monocytes did not show major differences in the reduction of active H3K4me3 histone marks. Alterations in epigenetic marks in HL60 cells upon differentiation are at par with our current understanding of epigenetic mechanisms.

*In vitro* cancer cell line model systems are extensively used to study molecular mechanisms, malignant phenotypes, drug-based studies, drug resistance, *etc.* They provide invaluable homogeneous tools for various experimental studies to obtain uniform and reproducible results (Drexler and MacLeod, 2003). Primary tumor cells/tissues are highly heterogeneous, difficult to culture, and exhibit experimental variability; as a result, multiple patient samples are required to obtain statistical strength. Hence, culture conditions, antibiotic usage, media formulations, and cell passage are essential to obtain uniform results (Ryu et al., 2017; Kurlbaum et al., 2020). Studies from different groups show that HL60 cells are cultured in RPMI or IMDM supplemented with 10% or 20% FBS, respectively. The American Type Culture Collection (ATCC) recommends the use of IMDM supplemented with 20% FBS for HL60 cell culture (https://www.atcc.org/products/ccl-240), while the European Collection of Authenticated Cell Cultures (ECACC) recommends RPMI 1640 + 2 mM glutamine supplemented with 10% FBS. Our results showed that the ATCC recommendation for HL60 culturing and myeloid differentiation is efficient compared to the ECACC recommendation. Moreover, studies have shown that the use of a variable percentage of FBS or different serum types brings variations in the outcome of the experimental results (Heger et al., 2018; Khasawneh et al., 2019). Higher calcium concentration in IMDM (1.49 mM) compared to RPMI medium (0.42 mM) could be yet an important factor that could have helped the optimal growth of HL60 cells. The optimal concentration of calcium ions is essential in signal transduction pathways, regulating cellular differentiation, proliferation, and apoptosis in a somatic cell (Carafoli, 2005). In addition, there are direct pieces of evidence that modulations in calcium concentration in the growth media can affect the growth and differentiation of the murine epidermal, corneal epithelial cells, keratinocytes, osteoblasts, and mesenchymal stem cells (Hennings et al., 1980; Bikle et al., 2012; Brauer et al., 2016; Aquino-Martinez et al., 2017; Masterton and Ahearne, 2019). It activates enzymes such as phosphodiesterases, protein kinases, and phosphorlipases, which are involved in cellular growth and differentiation (Zhu et al., 1993). Moreover, studies show that calcium concentration in the growth media affects the cellular proliferation and differentiation of leukemic cell lines such as HL60, KG-1, U937, and K562 cells where greater calcium influx correlates with differentiation (Okazaki et al., 1986; Rephaeli et al., 1990). Importantly, HL60 cells are well known for 10–12% spontaneous differentiation, so suboptimal growth conditions and media formulations may induce self-differentiation (Gallagher et al., 1979). Considering these factors, our study addressed an important aspect of culture condition uniformity for HL60 cells to obtain reproducible results.

Although cell line-based studies are useful and comparatively easy to conduct compared with primary cells, they often do not represent the *in vivo* situation at both the transcriptome and proteome levels (Lee et al., 2008; Pan et al., 2009). At the same time, working with primary cells present various challenges, such as procurement, purification, expensive media formulations, ethical considerations, and heterogeneity (Kaur and Dufour, 2012). Therefore, the usage of cell line-based research cannot be completely ignored. Rucker et al. performed molecular profiling of 17 myeloid leukemia cell lines and found stable molecular aberrations supporting the use of these cell lines as robust model systems (Rucker et al., 2006). Several studies were carried out in the 1980s, where researchers showed that HL60 cells can be effectively used for myeloid differentiation using various inducers. However, there are no studies available that compare HL60 cell line myeloid differentiation with normal differentiation. Our attempt to compare genome-wide transcriptome changes in HL60 vs GMP and vMono vs pMono revealed both similar and cell-specific gene expression profiles, which suggested that the myeloid differentiation process in primary cells is far more complex than *in vitro* myeloid differentiation. The complexity of *in vivo* myelopoiesis and limitations of *in vitro* HL60 myelopoiesis should be kept in mind when using HL60 cells. Nevertheless, HL60 myelopoiesis is well worked out, and our study supports its use to study myeloid differentiation.

Acute myeloid leukemia is a highly heterogeneous disease with multiple mutations and chromosomal translocations. AML patients can be treated with a combination of chemotherapeutic drugs that result in terminal differentiation and/or apoptosis of leukemic blasts. The HL60 model system has been used very effectively to study the effects of drugs and small molecules that can induce either differentiation and/or apoptosis (Huang et al., 2019). The functional role of genes of interest was also studied using RNAi strategies to understand their role in myeloid differentiation (Saha et al., 2022). In this regard, to validate the effectiveness of HL60 as an important leukemic cell line model, we compared the transcriptome of the uninduced HL60 cells and the primary leukemic blasts and obtained 68.03% of genes with a similar expression profile, which indicated a fair correlation between the uninduced HL60 cells and the leukemic blasts. The NSG category of genes showed pathways for metabolism, histone modification, transcript stability, *etc.*, which are essentially required for promoting cellular proliferation. Hence, we believe that HL60 cells can potentially be used to study the molecular mechanisms underlying AML. Furthermore, AML treatment regimens primarily focus on killing the cells or inducing proliferating cells to terminally differentiate, thereby reducing leukemic burden. HL60 cells show a systematic myeloid differentiation program under optimal culture conditions and therefore can be effectively used to study the role of various genes in reducing leukemic burden. We show that myeloid differentiation reduces the gene expression of several oncogenes, such as *MYC*, *FLT3*, *WT1*, and *KIT* suggesting that these gene subsets can be used as hallmarks to study the reduction in leukemic burden.

*In vitro* cell line models are very effective and popular among researchers for their homogeneity, long-term storage, revival, and availability across the globe compared with primary cells and therefore are invaluable resources for biomedical research (Drexler and MacLeod, 2003). Working with immortalized cells has limitations, as the experimental outcome may not directly translate to human physiology. Preclinical studies, such as drug-based studies, gene function studies, and toxicology studies carried out in vitro systems, must be carefully evaluated in organoid and animal model systems before obtaining conclusive results (Drexler and MacLeod, 2003; Wnorowski et al., 2019). Although we showed the effectiveness of the HL60 cell line model for various studies, the limitations of the *in vitro* model system are applicable to the current study. Our study showed the importance of using well-standardized cell culture methods and transcriptome changes associated with normal and malignant myelopoiesis. We showed both similarities and differences between primary myelopoiesis and *in vitro* myelopoiesis. The advantages and limitations of using HL60 as an *in vitro* model should be evaluated by researchers with careful experimental design and further validation, which should be carried out in *ex vivo* systems or in animal model systems.

## Data Availability

The data presented in the study are deposited in the EBI repository (https://www.ebi.ac.uk/biostudies/), accession number E-MTAB-12267.
