# Supplementary material for: Molecular and epigenetic alterations in normal and malignant myelopoiesis in human leukemia 60 (HL60) promyelocytic cell line model
Source: Front Cell Dev Biol. 2023 Feb 2;11:1060537. doi: 10.3389/fcell.2023.1060537 (PMC9932920; doi:10.3389/fcell.2023.1060537)
Supplement: Supplementary file 2 [file DataSheet1.docx]

**Molecular and epigenetic alterations in normal and malignant myelopoiesis in Human leukemia 60 (HL60) promyelocytic cell line model**

Basu, J., Madhulika, S. *et al.*

**Supplemental Appendix:**

**Contents**

1. **Supplemental Methods………………………………………………………...………….…...…..2**
   1. Cell Cycle Analysis.……………………………………………………….…………….……...…...2
2. **Supplemental results and Figures…………………………………………………………...…..2-9**

2.1. Myeloid differentiation induced in HL60 by Vit D3 and ATRA ………………………….…2-3

2.2 FACS sorting of the HL60 cells to obtain pure population for RNA-sequencing ………………4

2.3 Transcriptomic changes in HL60 cells post myeloid differentiation ..………………………..5-6

2.4 Comparative genome-wide transcription profile GMP vs HL60 and Vit D3 induced HL60 with primary monocytes …………………………………………………………………………...…...6-7

2.5 Optimization of induction of CD34+ HSPCs with M-CSF for generating monocytes………..7-8

2.6 Comparative transcriptomic between leukemic blast...………………………………………….9

1. **Table 1: List of differentially expressed epigenetic factors induced when HL60 cells were treated with Vit D3…………………………..….………………………………………………...10**
2. **Table 2: List of differentially expressed epigenetic factors induced when HL60 cells were treated with ATRA………..………………………………………………………………………11**
3. **Table 3: List of primer sequences used to validate the gene expression by qPCR.…………………………………………………………...……………………………...12-13**
4. **References…………………………………………………………………………………..…14-15**
5. **Supplemental methods**

**1.1 Cell Cycle Analysis**

Cell cycle analysis was performed for uninduced and induced HL60 cells in three biological replicates. 72 hours post differentiation, cells were harvested, washed with cold PBS, and fixed in 70 % ethanol for 2 hours on ice. The samples were then centrifuged at 1600 rpm for 5 min and washed in ice-cold PBS. The cell pellets were resuspended in 0.3 ml PBS containing 50 μg/ml propidium iodide (Himedia, TC252), 50 μg/ml RNase A (SIGMA, R4642), and 0.2 % Triton X-100 (SIGMA, T8787) and incubated for 15 mins at 37°C. The samples were acquired in FACS BD Accuri and data is analyzed using FlowJo Software version 10.7.1.**2.0**

**2.0 Supplemental results**

**2.1 Myeloid differentiation induced in HL60 by Vit D3 and ATRA (Related to Figure 1)**

To check the efficiency of myeloid differentiation induced in HL60 cells, we stimulated them with 50 nM vitamin D3 (Vit D3) and 10 µM *all-trans* retinoic acid (ATRA) for 72 hours to obtain monocytes and granulocytes respectively. The differentiated and undifferentiated HL60 cells were scored by flow cytometry analysis. The gating strategy used for segregating the cells was based upon their size scatter (SSC-A) and forward scatter (FSC-A) followed by selecting the single cells and finally scoring cells positive for CD14-APC-H7 and CD11b-FITC (Supplemental Figure S1A). Previous reports showed ATRA induction in HL60 arrests cell cycle cells possibly due to the upregulation of Apr3 (Apoptosis-related protein 3) which inhibits Cyclin D [1]. Also, ATRA induction ubiquitinates the cell cycle-related genes, promotes the cell to exit the cell cycle, and enhances differentiation in leukemic cells [2]. Therefore, we checked how the cell cycle got affected during myeloid differentiation in both IMDM and RPMI conditions. The cell cycle profile of cells cultured in IMDM media was assessed in uninduced, Vit D3, and ATRA-induced cells, where we observed a significant reduction in S-phase and G2-M arrest in the ATRA-induced cells compared with uninduced HL60 cells (Supplementary Figure 1B & D). In the Vit D3-induced HL60 cells, we found a G0-G1 arrest along with a significant reduction in the S-phase and G2-M phase suggesting an overall slowing of the cell cycle process. Next, we also performed cell cycle analysis for the RPMI conditions (Supplementary Figure 1C & D). There were similarities in the cell cycle profiles of uninduced and Vit D3 conditions in both the IMDM and RPMI conditions. However, contrary to the ATRA treatment in IMDM media, there was a reduction in the G2-M arrest in the RPMI condition indicating inadequate terminal differentiation of these cells. Thus, the cell cycle profiles of both IMDM and RPMI conditions corroborated well with the differentiation data mentioned. These results confirmed that the Vit D3 and ATRA induction significantly inhibited the cell cycle profiles of the HL60 cells. Overall, we showed efficient induction of myeloid differentiation of HL60 in the IMDM condition than the RPMI condition.


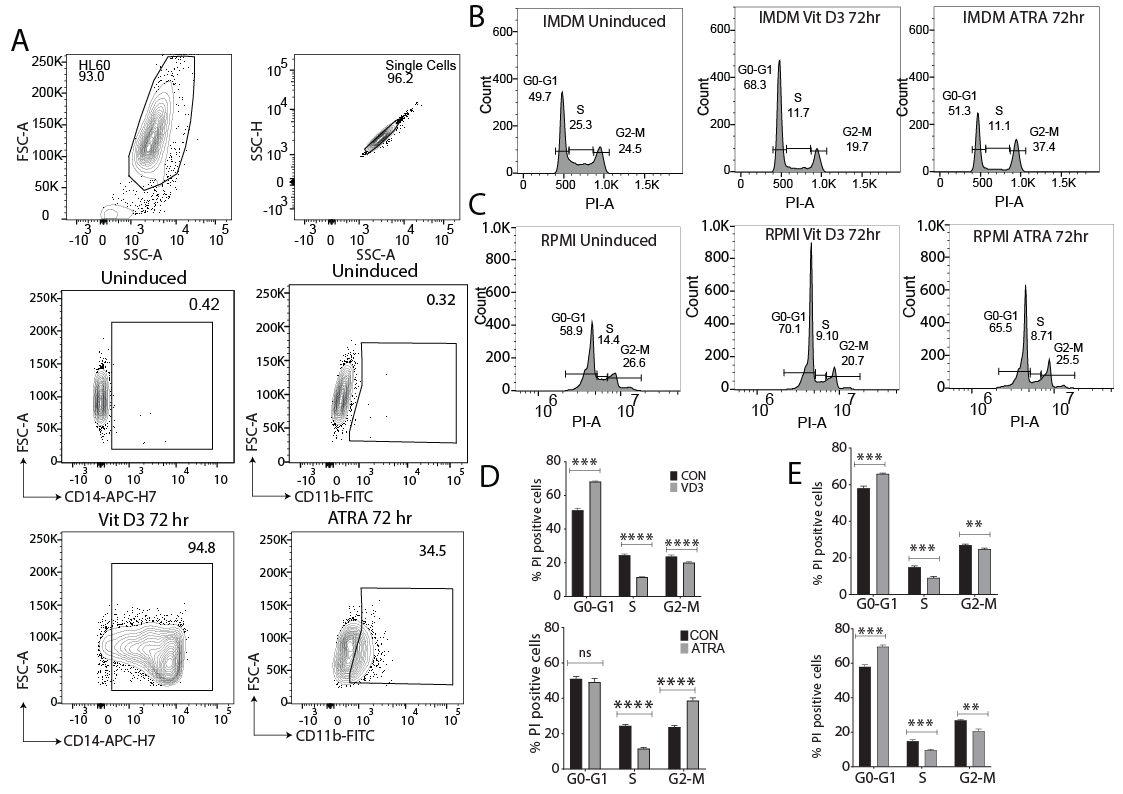


**Supplemental Figure S1: Myeloid differentiation in HL60 using Vitamin D3 (Vit D3) and *all-trans* retinoic acid (ATRA). (A)** Flow cytometry data showing contour plots with overall gating strategy applied for scoring for CD14-APC-H7 and CD11b-FITC positive HL60 cells post 72 hours of differentiation using Vit D3 and ATRA respectively in both IMDM and RPMI culturing conditions. The upper two contour plots show the gating based on the cell size (FSC-A), complexity (SSC-A) and selection of single cells. The middle and the lower panel shows representative contour plots of the monocyte (Vit D3) and granulocyte (ATRA) markers for CD14-APC H7 and CD11b-FITC in induced HL60 cells. **(B)** Representative histogram plots of the uninduced, Vit D3 and ATRA induced HL60 showing the changes in the cell cycle profile during the HL60 differentiation in IMDM medium. **(C)** Representative histogram plots of the uninduced, Vit D3 and ATRA induced HL60 showing the changes in the cell cycle profile during the HL60 differentiation in RPMI medium. **(D)** Bar plots showing the quantitation of number of cells present in G0-G1 (p = 0.000028), S-phase (p = 0.000008) and G2-M-phase (p = 0.004833) for Vit D3 and G0-G1 (p = 0.0.240382), S-phase (p = 0.000017) and G2-M-phase (p = 0.000130) for ATRA respectively in the IMDM condition. This experiment contains 6 biological and 3 technical replicates. **(E)** Bar plots showing the quantitation of number of cells present in G0-G1 (p = 0.000039), S-phase (p = 0.000072) and G2-M-phase (p = 0.001514) for Vit D3 and G0-G1 (p = 0.000033), S-phase (p = 0.000341) and G2-M-phase (p = 0.005632) for ATRA respectively in the RPMI condition. This experiment contains 5 biological and 3 technical replicates.

**2.2 FACS sorting of the HL60 cells to obtain pure population for RNA-sequencing**

HL60 cells cultured and differentiated with 50 nM Vit D3 and 10 µM ATRA in IMDM media were stained with CD14-APC-H7 and CD11b-FITC respectively. Stringent gating strategies were applied to sort cell populations that were either highly negative for CD11b and CD14 or highly positive for these markers. The gating of the uninduced cells was kept considerably tight to avoid the inclusion of any self-differentiated (mild CD11b-FITC and CD14-APCH7 positive) HL60 for the uninduced HL60 (Supplementary S2B). For sorting Vit D3-induced cells, we sorted cells that were highly positive for both CD11b and CD14. Similarly, for ATRA induction the cells were sorted that showed very high intensity of CD11b-FITC. (Supplementary S2C-D).


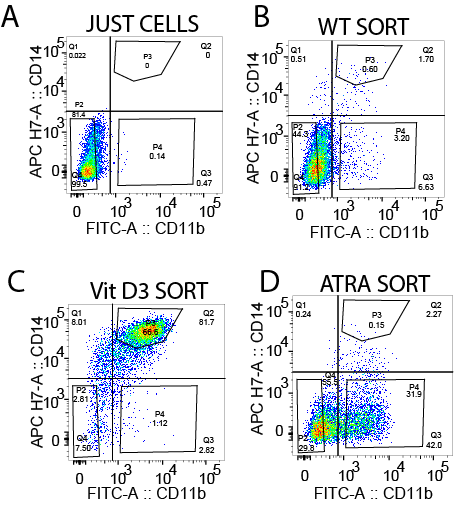


**Supplemental Figure S2: Gating Strategy applied for sorting pure populations of the uninduced and differentiated HL60 cells. (A)** Contour plot showing the gating of the unstained population. **(B)** Contour plot showing the gating for sorting pure population of the uninduced HL60, which were negative for CD11b-FITC and CD14-APC-H7. **(C)** Contour plot showing the gating for sorting pure population of the Vit D3 induced HL60 which were double-positive for CD11b-FITC and highly positive for CD14-APC-H7 and **(D)** Contour plot showing the gating for sorting pure population of the ATRA induced HL60 which were highly positive for CD11b-FITC and negative for CD14-APC-H7.

**2.3 Transcriptomic changes in HL60 cells post myeloid differentiation (Related to Figure 3)**

HL60 differentiation using Vit D3 and ATRA induction for 72 hours stimulated HL60 cells to differentiate into monocytes and granulocytes respectively. PCA plots generated from normalized read counts obtained from RNA-sequencing data showed similarity between the biological replicates (Supplemental Figure S3A). The differential gene expression analysis showed the total number of upregulated (n = 921 in Vit D3 & 1309 in ATRA) and downregulated (n = 225 in Vit D3 & 806 in ATRA) genes in the Vit D3 and ATRA induction respectively (Supplemental Figure S3B). The gene expression programs not only revealed the specific pathways related to immune function, and cell migration, typical of monocytes and neutrophils but also indicated initiation of terminal differentiation, for example, negative regulation of cell proliferation and positive regulation of cell death pathways were downregulated in Vit D3 and ATRA inductions. We observed pathways like Wnt/beta-catenin signaling in AML, cMYB pathway [3], transcriptional misregulation in cancer, and Ras signaling was downregulated in the Vit D3 monocytes (Supplemental Figure S3C). On similar lines, the downregulated pathways in the ATRA induction were associated with the MYC and CMYB pathways [3, 4], cell cycle [1], replication, and DNA repair [5], all of which are characteristic features of a terminally differentiated cell, which is granulocytes here (Supplemental Figure S3D). Thus, the transcriptomic data showed that the Vit D3 and ATRA induction not only elicited myeloid differentiation-specific pathways in the uninduced HL60 cells but also suppressed the oncogenic transcriptional programs.


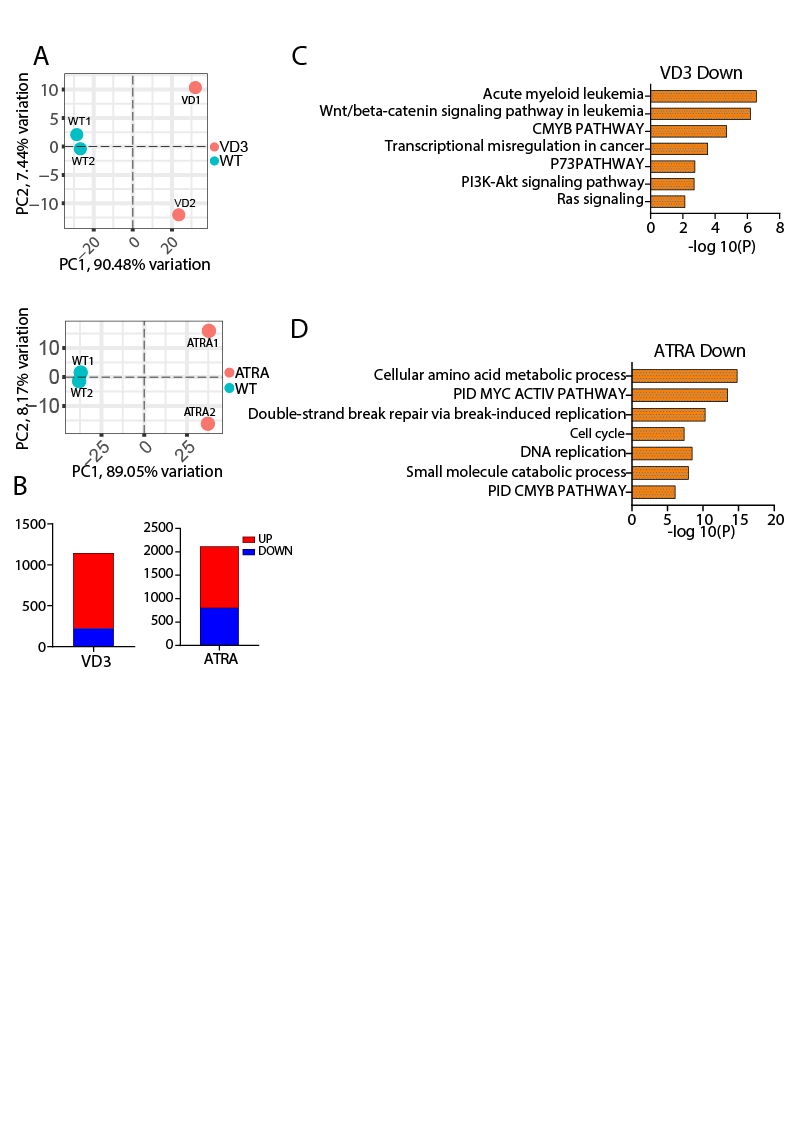


**Supplemental Figure S3: Differential gene expression analysis on uninduced and induced HL60 cells. (A)** Principal component analysis (PCA) showing the variance obtained from normalized read counts obtained from two biological replicates of the uninduced, Vit D3 (upper panel) and ATRA (lower panel) induced HL60 cells. **(B)** Stack plot representing the total numbers of the upregulated (n = 921 in Vit D3 & 1309 in ATRA) and the downregulated (n = 225 in Vit D3 & 806 in ATRA) genes obtained from Vit D3 and ATRA inductions in HL60. **(C)** Bar graphs showing significance (-log10 as p-value) for each pathways enriched from the downregulated genes obtained from Vit D3 induction compared with uninduced HL60 cells. **(D)** Bar graph showing significance (-log10 as p-value) for each pathways enriched from the downregulated genes obtained from ATRA induction compared with uninduced HL60 cells.

**2.4 Comparative genome-wide transcription profile GMP vs HL60 and Vit D3 induced HL60 with primary monocytes (Related to Figure 4)**

We showed that differential gene expression analysis between granulocyte-monocyte progenitor (GMP) and uninduced HL60 cell transcription profiles gave a significant number of genes with very low differential expression, which indicated that they possibly share common molecular signatures. In a similar analysis, we found Vit D3 induced HL60 to monocytes (vMono) and primary monocytes (pMono) share similar transcription profiles. In a similar analysis, we also found GMP-specific, HL60-specific, pMono-specific, and vMono-specific pathways. We observed that the upregulated genes (GMP-specific) were enriched for the hematopoietic cell lineage, regulation of cell adhesion, cytokine response, and metabolism (Supplemental Figure S4A). Interestingly, HL60-specific (downregulated genes) show mostly pathways related to different immune-responsive processes suggesting that HL60 cells are already tuned for immune function (Supplemental Figure S4B). The comparison between pMono and vMono revealed several immunological pathways like defense response to the virus, cytokine signaling, cell migration, and antigen processing and presentation were significantly enriched in pMono suggesting that they are better equipped to deal with the pathogen invasion (Supplemental Figure S4C). Contrary to this, vMono showed the enrichment of pathways related to the cell cycle, immune functions, cell migration, etc. (Supplemental Figure S4D). A higher concentration of Vit D3 or longer induction time may bring Vit D3-induced HL60 closer to functional pathways compared with pMono.


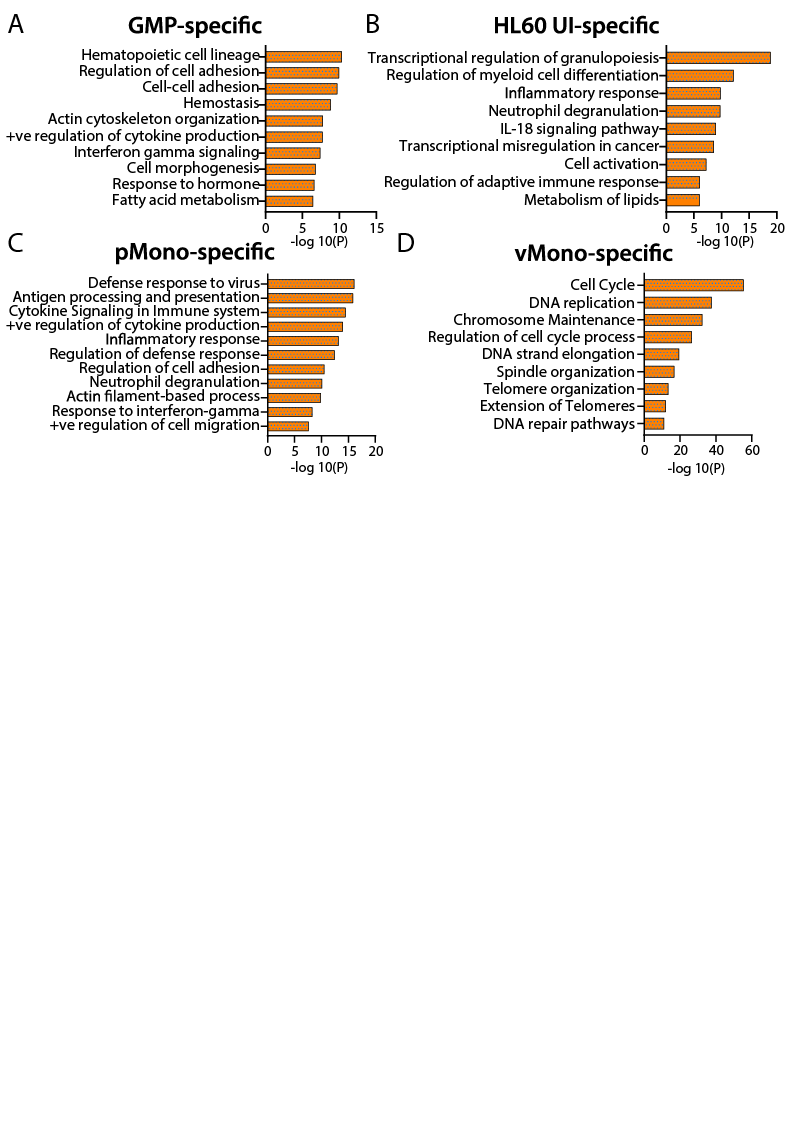


**Supplemental Figure S4: Upregulated and downregulated genes from GMP vs HL60 UI and pMono vs vMono transcriptome comparisons. (A)** Bar graph showing enriched pathways (significance -log10 as p-value) from upregulated genes in the GMP (GMP-specific) and **(B)** downregulated genes in uninduced HL60 cells (HL60-Specific). **(C)** Bar graph showing enriched pathways (significance -log10 as p-value) from upregulated genes in the peripheral monocytes (pMono-specific) and **(D)** downregulated genes in Vit D3 induced HL60 cells (vMono-specific).

**2.5 Optimization of induction of CD34+ HSPCs with M-CSF for generating monocytes**

**(Related to figure 4)**

In this study, we have used an ex vivo cord blood-derived CD34+ HSPCs model system for monocytic differentiation using M-CSF. M-CSF stimulated cord blood CD34+ HSPCs were differentiated into monocytes and HSPC marker CD34-PE-Cy7 and monocyte marker CD14-BV-605 were scored using flow cytometry to understand the extent of monocytic differentiation. The percentage of positive cells and MFI representing CD34+ and CD14+ cells before induction and after induction shows a significantly high number of CD34+ and CD14 positive cells respectively (Supplemental Figure S5A). This was further confirmed using May-Grünwald Giemsa staining which showed kidney-shaped nuclei post-M-CSF induction (Supplemental Figure S5B).


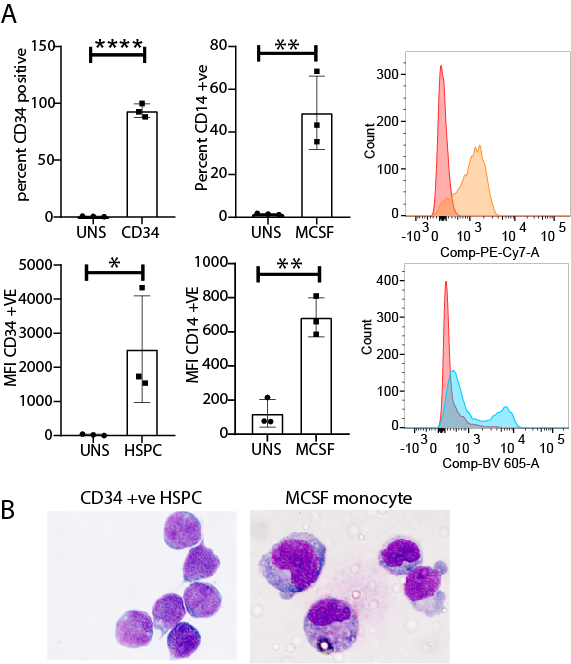


**Supplemental Figure S5. Ex vivo model system using cord blood CD34+ HSPCs for myeloid differentiation. (A)** Bar plots representing the levels of percent positive cells (upper panel) and the MFI intensity (lower panel) of the CD34-PE-Cy7 and CD14-BV-605 in the ex vivo differentiation system. The CD34-PE-Cy7 levels were checked in the CD34 positive HSPCs and the CD14-BV-605 levels were checked in the M-CSF induced HSPCs (** = 0.01, *** = 0.001, **** = 0.0001). Histogram showing the CD34 levels before induction (upper panel) and CD14 levels after M-CSF induction (lower panel) **(B)** May Grünwald Giemsa staining showing the nuclear morphology changes upon ex vivo HSPC differentiation into monocytes and granulocytes upon M-CSF stimulation. The image is taken in Olympus IX83 inverted microscope using CellSens imaging software (Olympus).

**2.6 Comparative transcriptomic between leukemic blast cells and HL60 cells (Related to Figure 5)**


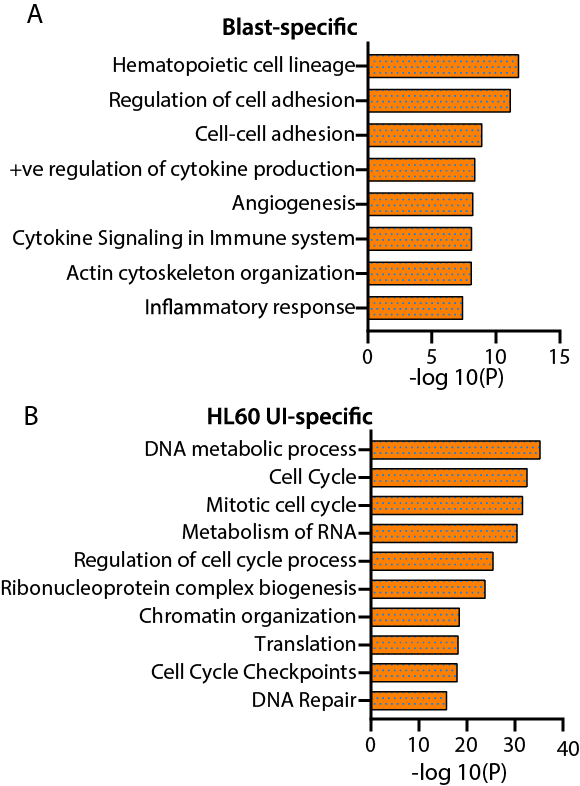
Differential gene expression analysis between the uninduced HL60 and the primary AML blast cell showed a sufficient gene expression overlap. However, we obtained the upregulated genes (blast-specific) that potentially contribute to the uniqueness of the AML blast. Pathways associated with this gene subset showed enrichment of cell lineage, cell adhesion, regulation by cytokines, angiogenesis, and inflammatory response (Supplemental Figure S6A). The unlimited proliferation in AML blast is facilitated by IL1b, IL6, and IL8 cytokines that result in a chronic inflammatory response [6]. Several chromosomal translocations in AML are reported to induce cell adhesion molecules [7] apart from an enhanced level of bone marrow angiogenesis in the leukemic blast cells [8]. On the contrary, the downregulated genes (HL60-specific) were enriched for the regulation of the cell cycle. This is true for blast cells which due to their leukemic transformation undergo deregulation in their cell cycle checkpoints[9]. This results in their uncontrolled proliferative capacity with compromised differentiation ability. Also, we observed processes like DNA repair and chromatin organization were down which is evident in the AML cells that accumulate several mutations higher in the hematopoietic lineage (Supplemental Figure S6B)

**Supplemental Figure S6: Pathway enrichment analysis for upregulated (blast-specific) and downregulated (HL60-specific) genes. (A)** Bar graph showing enriched pathways (significance -log10 as p-value) from upregulated genes in the AML primary blast cells compared with uninduced HL60 cells. **(B)** Bar graph showing enriched pathways (significance -log10 as p-value) from downregulated (HL60-specific) genes in the AML primary blast cells compared with uninduced HL60 cells.

**Table 1: List of differentially expressed epigenetic factors induced when HL60 cells were treated with Vit D3.**

| **Gene name** | **Function** | **References** |
| --- | --- | --- |
| *ZBTB16* | CD34 positive progenitor-specific factor that reduces upon differentiation and it reduces with oncogenic transformations. | [10] |
| *TET1* | Highly expressed in cytogenetically normal (CN) AML cases specifically in M0/M1 phenotype and with NPM1 mutation. Its over-expression is associated with poor overall survival of CN AML patients. | [11] |
| *BAHCC1* | Recognizes the repressive H3K27me3 histone mark and is over-expressed in AML condition and its knockdown de-represses tumor suppressors and induces differentiation in the leukemic cells. | [12] |
| *BRPF1* | HSC-specific chromatin remodeler (reader) and in AML, it regulates the self-renewal property essential for leukemogenesis. | [13] |
| *APOBEC3A* | Highly expressed in pediatric and adult AML, which can be targeted to induce apoptosis through ATR inhibitors. | [14] |
| *PHC2* | An important epigenetic factor that regulates the HSPC traffic through the removal of repression of VCAM1. | [15] |
| *SIN3B* | An HSC-specific factor that regulates its quiescence and proliferation and facilitates differentiation and protection against hematopoietic injury. | [16] |
| *PADI4* | Associated with autoimmunity and cancer and its expression is restricted to bone marrow. It regulates hematopoietic self-renewal, differentiation, and regeneration. | [17] |
| *TDRD9* | Highly expressed in ~50% of acute myeloid tumors. | [18] |
| *HIF3A* | In Zebrafish studies, HIF3A is found to regulate erythropoiesis through GATA1. | [19] |
| *PHC1* | Facilitates PRC1 complex functioning in hematopoiesis and B cell development. | [20] |
| *BAZ2B* | Along with other transcription factors, it forms a network that is implicated in promoting stem properties and multipotency in the HSPCs and regulates distal regulatory chromatin in the committed progenitors. | [21] |

**Table 2: List of differentially expressed epigenetic factors induced when HL60 cells were treated with ATRA.**

| **Gene name** | **Function** | **Reference** |
| --- | --- | --- |
| *RUVBL2* | Essential for Acute Myeloid Leukemia by targeting the c-MYB target gene that is required for normal myeloid differentiation. Targeting RUVBL2 however, did not have any effect on normal hematopoiesis. | [22] |
| *PRMT1* | High levels of PRMT1 along with FLT3-ITD contribute to the maintenance of AML. Increased methylation of FLT3 by PRMT1 aids in the process. | [23, 24] |
| *NCOA3* | In-situ FISH detected fusion of MYST3-NCOA3 in M4/M5 AML patient samples. | [25] |
| *KAT2A* | Essential for the development of leukemia in mice. Loss of KAT2A promotes the leukemic cells towards maturation and loss of proliferation. | [26] |
| *PRMT5* | Important for leukemia development by increasing leukemic differentiation block. | [27] |
| *SMYD5* | SMYD5 knockout zebrafishes showed increased hematopoietic markers like PU.1 | [28] |
| *CBX7* | Upregulated and facilitates self-renewal in the AML condition. Its depletion promotes differentiation and reduced proliferation in the AML cells. | [29] |
| *DNMT3A* | Mutated up to 20% of cases in AML patients with poor overall survival. | [30] |
| *MECOM* | Over-expressed in the pediatric AML and can be used as a prognostic marker for patients with no previously detected cytogenetic risk. | [31] |
| *NPM1* | Mutated in ~30% of the AML cases, often in association with FLT3 and in the cytoplasm of the AML cell, the mutated NPM1 is delocalized. | [32] |

**Table 3: List of primer sequences used to validate the gene expression by qPCR.**

| **Gene Name** | **Primer Sequence** |
| --- | --- |
| *PD1-L1/CD274*_F | TGGCATTTGCTGAACGCATTT |
| *PD1-L1/CD274*_R | TGCAGCCAGGTCTAATTGTTTT |
| *RSAD2*-F | CCAGTGCAACTACAAATGCGGC |
| *RSAD2*-R | CGGTCTTGAAGAAATGGCTCTCC |
| *NFKBIA*-F | ACCTGGTGTCACTCCTGTTGA |
| *NFKBIA*-R | CTGCTGCTGTATCCGGGTG |
| *MSR1*-F | GCAGTGGGATCACTTTCACAA |
| *MSR1*-R | AGCTGTCATTGAGCGAGCATC |
| *EDN1*-F | TCTCTGCTGTTTGTGGCTTG |
| *EDN1*-R | GACTGGGAGTGGGTTTCTCC |
| *KLF4*-F | GCGCGTTCCTTACTTATAACTTCC |
| *KLF4*-R | TAGCAACGATGGAAGGGAGC |
| *LYN*-F | GCGTGCTCAAGCTCATGTTC |
| *LYN*-R | GAAGTGGGTATCCACGGTGC |
| *ICAM1*-F | GTATGAACTGAGCAATGTGCAAG |
| *ICAM1*-R | GTTCCACCCGTTCTGGAGTC |
| *HCK*-F | CACGAAGACCTCAGCTTCCAG |
| *HCK*-R | GATCGAGCCTTCCACCACT |
| *CCL3*-F | GGCTCTCTGCAACCAGTTCT |
| *CCL3*-R | TGAAATTCTGTGGAATCTGCC |
| *IRF1*-F | CATGAGACCCTGGCTAGAGATG |
| *IRF1*-R | TCCGGAACAAACAGGCATCC |
| *AQP9*-F | TGTCTCTTTGGACGGATGAAATG |
| *AQP9*-R | TCTCCCACGATCAGCAGTTTT |
| *CD9*-F | CCTGCTGTTCGGATTTAACTTCA |
| *CD9*-R | TGGTCTGAGAGTCGAATCGGA |
| *CD37*-F | TCCTGAGAGGTAACGGGTCG |
| *CD37*-R | GGATTGTGGAGTCGTTGGTCG |
| *FN1*-F | AGGAAGCCGAGGTTTTAACTG |
| *FN1*-R | AGGACGCTCATAAGTGTCACC |
| *IL4R*-F | ACACCAATGTCTCCGACACTC |
| *IL4R-*R | TGTTGACTGCATAGGTGAGATGA |
| *KIT*-F | ACTTGAGGTTTATTCCTGACCCC |
| *KIT*-R | GCAGACAGAGCCGATGGTAG |
| *FLT3*-F | AGGGACAGTGTACGAAGCTG |
| *FLT3*-R | GCTGTGCTTAAAGACCCAGAG |
| *WT1*-F | TGCTTACCCAGGCTGCAATAA |
| *WT1*-R | TTCTCACCAGTGTGCTTCCTG |
| *GATA2*-F | ACTGACGGAGAGCATGAAGAT |
| *GATA2*-R | CCGGCACATAGGAGGGGTA |
| *PRMT1*-F | TACTACTTTGACTCCTATGCCCA |
| *PRMT1*-R | ATGCCGATTGTGAAACATGGA |
| *PRMT5*-F | CTGAATTGCGTCCCCGAAATA |
| *PRMT5*-R | AGGTTCCTGAATGAACTCCCT |
| *DNMT3A*-F | TATTGATGAGCGCACAAGAGAGC |
| *DNMT3A*-R | GGGTGTTCCAGGGTAACATTGAG |
| *KAT2A*-F | CAGGGTGTGCTGAACTTTGTG |
| *KAT2A*-R | TCCAGTAGTTAAGGCAGAGCAA |
| *NCOA3*-F | ACATAAACGCCAGTCCTGAAAT |
| *NCOA3*-R | CCTTCCTCCATCATAGCTCGT |
| *IDH2*-F | CGCCACTATGCCGACAAAAG |
| *IDH2*-R | ACTGCCAGATAATACGGGTCA |
| *SMC1A*-F | CATCAAAGCTCGTAACTTCCTCG |
| *SMC1A-*R | CCCCAGAACGACTAATCTCTTCA |

**3.0 References**

1. Yu, F., et al., *Apoptosis related protein 3, an ATRA-upregulated membrane protein arrests the cell cycle at G1/S phase by decreasing the expression of cyclin D1.* Biochem Biophys Res Commun, 2007. **358**(4): p. 1041-6.

2. Fang, Y., et al., *The ubiquitin-proteasome pathway plays essential roles in ATRA-induced leukemia cells G0/G1 phase arrest and transition into granulocytic differentiation.* Cancer Biol Ther, 2010. **10**(11): p. 1157-67.

3. Walf-Vorderwulbecke, V., et al., *Targeting acute myeloid leukemia by drug-induced c-MYB degradation.* Leukemia, 2018. **32**(4): p. 882-889.

4. Birnie, G.D., *The HL60 cell line: a model system for studying human myeloid cell differentiation.* Br J Cancer Suppl, 1988. **9**: p. 41-5.

5. Nouspikel, T. and P.C. Hanawalt, *DNA repair in terminally differentiated cells.* DNA Repair (Amst), 2002. **1**(1): p. 59-75.

6. Hemmati, S., T. Haque, and K. Gritsman, *Inflammatory Signaling Pathways in Preleukemic and Leukemic Stem Cells.* Front Oncol, 2017. **7**: p. 265.

7. Roselova, P., et al., *Adhesion structures in leukemia cells and their regulation by Src family kinases.* Cell Adh Migr, 2018. **12**(3): p. 286-298.

8. Testa, U., G. Castelli, and E. Pelosi, *Angiogenesis in acute myeloid leukemia.* Journal of Cancer Metastasis and Treatment, 2020. **6**: p. 53.

9. Didier, C., et al., *G2/M checkpoint stringency is a key parameter in the sensitivity of AML cells to genotoxic stress.* Oncogene, 2008. **27**(27): p. 3811-3820.

10. Suliman, B.A., D. Xu, and B.R. Williams, *The promyelocytic leukemia zinc finger protein: two decades of molecular oncology.* Front Oncol, 2012. **2**: p. 74.

11. Wang, J., et al., *High Expression of TET1 Predicts Poor Survival in Cytogenetically Normal Acute Myeloid Leukemia From Two Cohorts.* EBioMedicine, 2018. **28**: p. 90-96.

12. Fan, H., et al., *BAHCC1 binds H3K27me3 via a conserved BAH module to mediate gene silencing and oncogenesis.* Nat Genet, 2020. **52**(12): p. 1384-1396.

13. You, L., et al., *BRPF1 is essential for development of fetal hematopoietic stem cells.* J Clin Invest, 2016. **126**(9): p. 3247-62.

14. Green, A.M., et al., *Cytosine Deaminase APOBEC3A Sensitizes Leukemia Cells to Inhibition of the DNA Replication Checkpoint.* Cancer Res, 2017. **77**(17): p. 4579-4588.

15. Bae, J., et al., *Phc2 controls hematopoietic stem and progenitor cell mobilization from bone marrow by repressing Vcam1 expression.* Nat Commun, 2019. **10**(1): p. 3496.

16. Cantor, D.J. and G. David, *The chromatin-associated Sin3B protein is required for hematopoietic stem cell functions in mice.* Blood, 2017. **129**(1): p. 60-70.

17. Young, C., et al., *Intrinsic function of the peptidylarginine deiminase PADI4 is dispensable for normal haematopoiesis.* Biol Open, 2022. **11**(6).

18. Guijo, M., et al., *Expression of TDRD9 in a subset of lung carcinomas by CpG island hypomethylation protects from DNA damage.* Oncotarget, 2018. **9**(11): p. 9618-9631.

19. Cai, X., et al., *Zebrafish Hif3alpha modulates erythropoiesis via regulation of gata1 to facilitate hypoxia tolerance.* Development, 2020. **147**(22).

20. Di Carlo, V., I. Mocavini, and L. Di Croce, *Polycomb complexes in normal and malignant hematopoiesis.* J Cell Biol, 2019. **218**(1): p. 55-69.

21. Arumugam, K., et al., *The Master Regulator Protein BAZ2B Can Reprogram Human Hematopoietic Lineage-Committed Progenitors into a Multipotent State.* Cell Rep, 2020. **33**(10): p. 108474.

22. Armenteros-Monterroso, E., et al., *The AAA+ATPase RUVBL2 is essential for the oncogenic function of c-MYB in acute myeloid leukemia.* Leukemia, 2019. **33**(12): p. 2817-2829.

23. He, X., et al., *PRMT1-mediated FLT3 arginine methylation promotes maintenance of FLT3-ITD(+) acute myeloid leukemia.* Blood, 2019. **134**(6): p. 548-560.

24. Zhu, L., et al., *Protein arginine methyltransferase 1 is required for maintenance of normal adult hematopoiesis.* Int J Biol Sci, 2019. **15**(13): p. 2763-2773.

25. Esteyries, S., et al., *NCOA3, a new fusion partner for MOZ/MYST3 in M5 acute myeloid leukemia.* Leukemia, 2008. **22**(3): p. 663-5.

26. Domingues, A.F., et al., *Loss of Kat2a enhances transcriptional noise and depletes acute myeloid leukemia stem-like cells.* Elife, 2020. **9**.

27. Tarighat, S.S., et al., *The dual epigenetic role of PRMT5 in acute myeloid leukemia: gene activation and repression via histone arginine methylation.* Leukemia, 2016. **30**(4): p. 789-99.

28. Fujii, T., et al., *Smyd5 plays pivotal roles in both primitive and definitive hematopoiesis during zebrafish embryogenesis.* Sci Rep, 2016. **6**: p. 29157.

29. Jung, J., et al., *CBX7 Induces Self-Renewal of Human Normal and Malignant Hematopoietic Stem and Progenitor Cells by Canonical and Non-canonical Interactions.* Cell Rep, 2019. **26**(7): p. 1906-1918 e8.

30. Park, D.J., et al., *Characteristics of DNMT3A mutations in acute myeloid leukemia.* Blood Res, 2020. **55**(1): p. 17-26.

31. Elsherif, M., et al., *MECOM gene overexpression in pediatric patients with acute myeloid leukemia.* Acta Oncol, 2022. **61**(4): p. 516-522.

32. Falini, B., et al., *Diagnostic and therapeutic pitfalls in NPM1-mutated AML: notes from the field.* Leukemia, 2021. **35**(11): p. 3113-3126.
